# Human mobility and the spatial transmission of influenza in the United States

**DOI:** 10.1371/journal.pcbi.1005382

**Published:** 2017-02-10

**Authors:** Vivek Charu, Scott Zeger, Julia Gog, Ottar N. Bjørnstad, Stephen Kissler, Lone Simonsen, Bryan T. Grenfell, Cécile Viboud

**Affiliations:** 1 Fogarty International Center, National Institutes of Health, Bethesda, MD, United States of America; 2 Department of Biostatistics, Johns Hopkins Bloomberg School of Public Health, Baltimore, MD, United States of America; 3 Department of Applied Mathematics and Theoretical Physics, University of Cambridge, Cambridge, United Kingdom; 4 Department of Entomology, Pennsylvania State University, State College, Pennsylvania, United States of America; 5 Department of Public Health, Copenhagen University, Copenhagen, Denmark; 6 Department of Ecology and Evolutionary Biology, Princeton University, Princeton, NJ, United States of America; Ecole Polytechnique Federale de Lausanne, SWITZERLAND

## Abstract

Seasonal influenza epidemics offer unique opportunities to study the invasion and re-invasion waves of a pathogen in a partially immune population. Detailed patterns of spread remain elusive, however, due to lack of granular disease data. Here we model high-volume city-level medical claims data and human mobility proxies to explore the drivers of influenza spread in the US during 2002–2010. Although the speed and pathways of spread varied across seasons, seven of eight epidemics likely originated in the Southern US. Each epidemic was associated with 1–5 early long-range transmission events, half of which sparked onward transmission. Gravity model estimates indicate a sharp decay in influenza transmission with the distance between infectious and susceptible cities, consistent with spread dominated by work commutes rather than air traffic. Two early-onset seasons associated with antigenic novelty had particularly localized modes of spread, suggesting that novel strains may spread in a more localized fashion than previously anticipated.

## Author summary

The underlying mechanisms dictating the spatial spread of seasonal influenza remain poorly understood, in part because of the lack of spatially resolved disease data to quantify patterns of spread. In this paper, we address this issue by analyzing fine-grain insurance claims data on influenza-like-illnesses over eight seasons in ~300 locations throughout the United States. Using statistical methods, we found that seven of eight epidemics likely originated in the Southern US, that influenza spatial transmission is dominated by local traffic between cities, and that seasons marked by novel influenza virus circulation had a particularly radial, localized spatial structure. These findings are in stark contrast to prevailing theories of influenza spatial transmission that suggest that transmission is favored in low humidity environments and that spread is a dominated by air traffic between populous hubs.

## Introduction

Understanding the spatial spread of infectious diseases is essential for clarifying mechanisms of transmission and targeting control interventions. Seasonal influenza offers a unique opportunity to study the spatial diffusion of a directly transmitted pathogen in partially immune populations due the yearly invasion, extinction and subsequent re-invasion of viral strains in the northern and southern hemispheres [1–4]. Detailed characterization of the underlying mechanisms of spread has, however, been hindered by lack of spatially resolved incidence data spanning multiple seasons [5].

A number of studies have explored the roles of human mobility, demography, and environmental factors in the spread of seasonal influenza on global and regional scales. These studies have suggested the following “principles”: (i) at a global scale, the worldwide air transportation network serves as the predominant channel for the dissemination of pandemic and seasonal influenza viruses, A/H3N2 viruses in particular [3,6–11]; (ii) at the regional scale of the US, short-distance work commutes are a major driver of the spread of seasonal outbreaks, though longer-range air traffic has been implicated as well [1,12–14]; (iii) influenza is marked by rapid, hierarchical spread between populous centers followed by subsequent spread to less populated areas [1]; (iv) low-humidity environments favor viral stability and thus transmission of influenza [15,16]. These sometimes-conflicting findings highlight the complexity of influenza spatial transmission across geographic scales [5] and indicate that more detailed analyses are necessary to deepen our understanding of the drivers of spread.

Though rare, influenza pandemics offer valuable opportunities to study the dissemination of an invasion wave in a susceptible population, with higher than usual attack rates and intensified data collection efforts. Previous work has indicated that gravity models [17] can provide parsimonious descriptions of the spatial diffusion process of pandemic waves, particularly for the 1918 pandemic in England and Wales [18], and the 2009 A/H1N1pdm pandemic in the US [19]. These models describe the pairwise spatial interaction between communities as a function of population size and distance, each tuned by power-law parameters. A previous analysis of the 2009 pandemic across the continental US revealed a surprisingly local and radially diffusive wave of spread originating in the Southeastern US [19]. The observed pandemic trajectory ran contrary to the prediction of fast hierarchal invasion among populous and interconnected locales followed by slower “in-filling” of the interspersed smaller communities. We currently have no guiding principles for when or whether we should expect such a localized mode of spread for seasonal epidemics since there are substantial differences in both the age-groups infected and in population-level immunity during epidemic v. pandemic seasons [20]. Further, prior work has indicated that global and regional patterns of influenza spread and viral persistence differ by subtype, perhaps mediated by differenced in immunity [1,4]. Within a subtype, prior exposure history and antigenic changes may render specific age-strata differentially immune to the particular viral strain in circulation, in turn affecting patterns of spread. In the absence of a full understanding of the interplay between prior immunity and disease dissemination, mobility studies and simulation models must be validated with real-world incidence data.

Here, we present a detailed analysis of influenza spread between US cities by modeling a unique set of geo-referenced time series of influenza-like illnesses generated from a large-volume compilation of outpatient medical insurance claims across the US, spanning 8 influenza seasons. Our analysis provides the first detailed characterization of seasonal influenza spread across the continental US and its relation to subtype-differences in susceptibility/contagiousness, human mobility and environmental factors.

## Results

**Fig 1:**
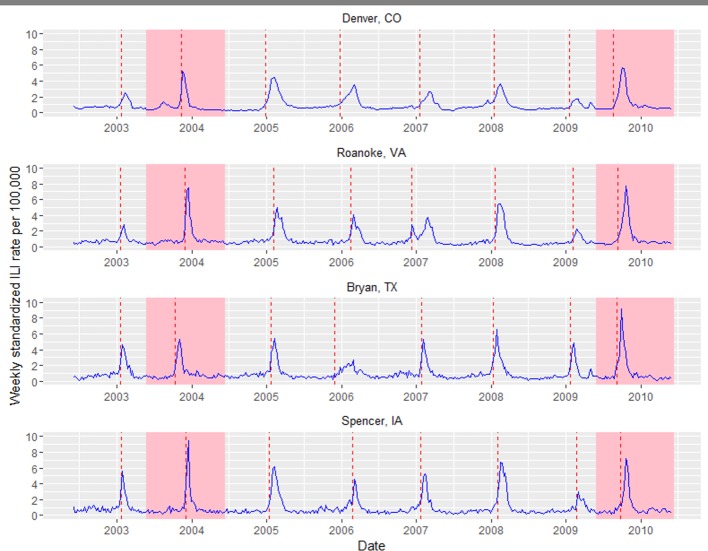
Standardized influenza-like-illness time series from four locations (Denver, CO; Roanoke, VA; Bryan, TX; Spencer, IA). Onset times for each season are depicted as red dashed lines. The 2003/2004 epidemic and 2009/2010 pandemic are highlighted in pink as these are major influenza seasons associated with antigenic novelty, where more than 99% of viruses in circulation were of the same subtype (A/H3N2 in 2003/2004 and A/H1N1 in 2009/2010). These major seasons had the most distinct influenza signals, while few locations experienced an increase in ILI during the mild 2008/2009 epidemic dominated by the A/H1N1 virus.

Our analysis builds on earlier work indicating that city-level medical insurance claims of influenza-like-illnesses (ILI) are useful and specific indicators of influenza virus activity across the US [21]. We extend previous analyses of the autumn wave of the 2009 A/H1N1 pandemic [19] to study spatial spread among 310 distinct geo-referenced locations across continental US during the 2002/03 through 2009/10 influenza seasons, covering both mild and severe epidemics as well as the 2009 pandemic (Table 1, supporting S1 Text, Table A1). Our spatial analysis relies on estimates of local influenza onsets, defined as the date associated with the winter breakpoint in ILI incidence for each location in each season (see methods, Fig 1 and supporting S1 Text, Fig A1). Depending on the strength of the influenza signal, we were able to accurately estimate the onset times in 135–306 locations in each season (Table 1). First, we characterize the timing, origin, trajectory, spatial synchrony and long-range transmission events of each of the 8 epidemics. We subsequently fit power-law gravity models with and without explicit mobility indicators to the incidence data.

**Table 1:** Virological and epidemiological characteristics of US influenza epidemics, 2002–2010.

Season	Dominant Subtype	Antigenic characteristics	Age distribution (% 5yrs)	Cities (#)	Time for spread* (weeks)	Calendar time**	Long-range events*** (#)
**2002/2003**	A/H1N1 + B	CAL99 (A/H1N1) SY97 (A/H3N2)	69.1%	233	10.22 (6.93; 16.25)	Dec.-Jan.	2
**2003/2004**	A/H3N2	FU02 (A/H3N2)	67.9%	306	11.94 (4.89; 14.00)	Oct.-Nov.	1
**2004/2005**	A/H3N2 + B	CA04 (A/H3N2)	48.2%	285	9.76 (6.27; 12.80)	Dec.-Jan.	5
**2005/2006**	A/H3N2 + B	CA04 (A/H3N2)	59.0%	232	13.17 (10.88; 22.39)	Nov.-Feb.	2
**2006/2007**	A/H1N1 + B	CAL99 (A/H1N1) WI05 (A/H3N2)	70.8%	208	14.43 (9.25; 20.19)	Nov.-Jan.	3
**2007/2008**	A/H3N2 + B	WI05 (A/H3N2)	55.7%	290	10.35 (3.82; 12.78)	Dec.-Jan.	3
**2008/2009**	A/H1N1 + B	BR07 (A/H1N1) WI05 (A/H3N2)	71.6%	135	4.43 (4.55; 10.63)	Jan.-Feb.	2
**2009/2010**	A/H1N1pdm	CA09 (A/H1N1pdm) PE09 (A/H3N2)	62.5%	303	8.48 (8.22, 12.35)	Aug.-Oct.	2

Dominant subtype indicates the subtype representing >50% of circulating viruses in a given season. Antigenic characteristics are based on [40–42]; underlined antigenic clusters represent the dominant influenza strain (if any). The 5^th^ column indicates the number of cities for which accurate epidemic onsets could be estimated, out of a total of 310 cities in the dataset.

*Time for 50% of cities to be infected (Time from the 5^th^ percentile of cities infected to the 95^th^ percentile of cities infected; Time from the first city to be infected to the last city to be infected).

**Calendar time for 90% of cities to be infected (5^th^ percentile to the 95^th^ percentile).

*** Above ~940km; see main text for details. See supporting S1 Text, Table A2 for additional thresholds for defining the number of long-range transmission events.

### Temporal and spatial patterns in city-level influenza onset times

**Fig 2:**
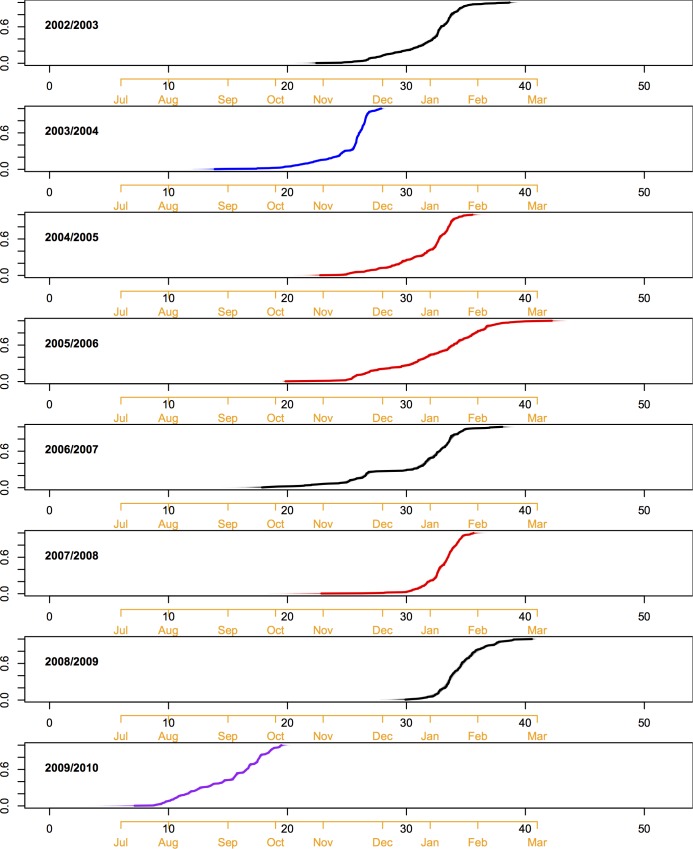
Cumulative epidemic curves display the proportion of locations infected over time in each season. In each panel, the x-axis represents weeks since June 1^st^, with the corresponding calendar months labeled in orange. Each graph represents 100 possible epidemic curves generated by varying local onset times within estimated bounds of uncertainty. The tightness of the curves indicates that uncertainty in local onset times has little effect on the epidemic’s overall trajectory. Epidemic curves are colored according to the dominant subtype in circulation (black corresponds to A/H1N1 + B, blue to A/H3N2, red to A/H3N2 + B, and purple to A/H1N1pdm).

Fig 2 depicts the proportion of infected locations over time in each season, illustrating variability in both the epidemic period and the shape of the epidemic curves. The time for influenza to reach all US locations ranged from ~11–22 weeks, while the time for 90% of locations to be infected had a narrower, but still highly variable range (~5–11 weeks, based on the 5^th^ to 95^th^ percentiles, Table 1). Across seasons, onset times typically clustered between November and January, with two notable exceptions associated with major influenza seasons in 2003/2004 (Oct.-Nov.) and the 2009 pandemic (Aug.-Oct.). Epidemic curves were generally sigmoidal, indicating that the majority of transmission events happen over a narrow time interval relative to the entire length of the epidemic (Fig 2). Notable exceptions were the 2005/2006 A/H3N2 season and the 2009 pandemic, both of which had a fairly linear and steady progression of cities becoming infected, and the 2006/2007 A/H1N1+B epidemic which appears to have two “waves” of transmission (Fig 1, Panel 2 and Fig 2).

**Fig 3:**
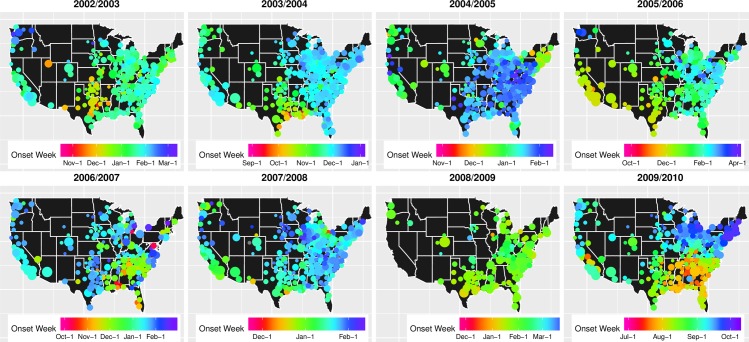
Influenza epidemic onset times across eight seasons, 2002/2003-2009/2010 (as estimated based on outpatient influenza-like-illness time series). Each circle represents a distinct location, and the size of the circle is proportional to the population size of the location. The relative ordering of influenza onsets is depicted in color, magenta indicating locations with earliest onsets and purple indicating locations with latest onsets.

**Fig 4:**
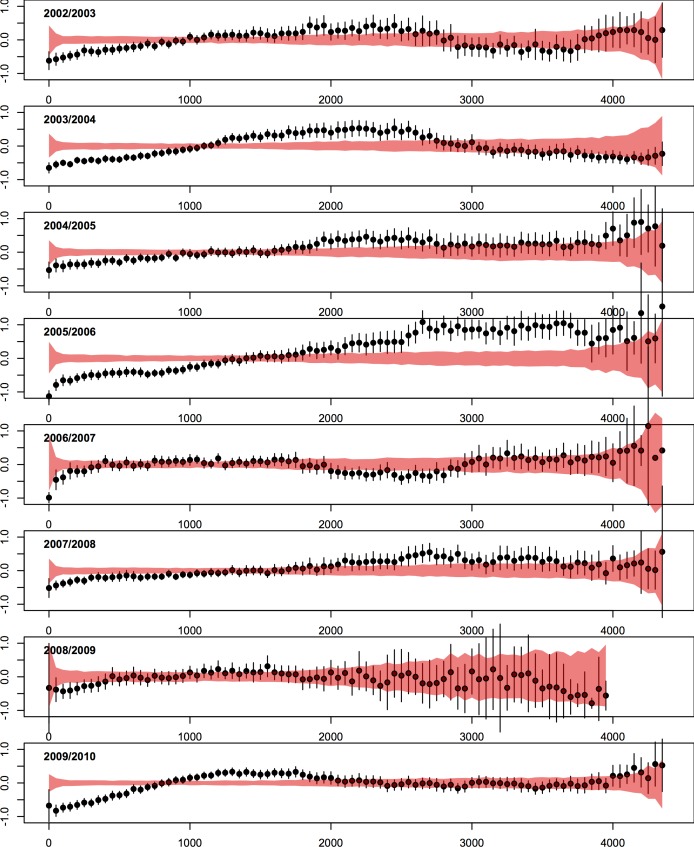
Pairwise synchrony as a function of pairwise distance in each influenza season (2002/2003–2009/2010). The y-axis in each panel is a normalized measure of pairwise synchrony in onset times between locations (values near -1 indicate that epidemics start close together in time, while values near +1 indicate a substantial lag in onset times). The x-axis is distance (kilometers). Each circle represents the mean pairwise synchrony for pairs of locations falling in 50-km distance bins. The black line segments are 95% confidence intervals for the mean in each bin. The red band is the expected pairwise synchrony under the null hypothesis of complete spatial randomness obtained by permutation of the onset times.

Maps of estimated influenza onset times reveal clear spatial patterns in all seasons (Fig 3 and supporting S1 Text, Figure A2). Marked radial patterns are observed in at least four seasons: 2002/2003 (the hub of the epidemic is in the Southern US, see also supporting S1 Text, Figure A4), 2003/2004 (Southern US), 2005/2006 (Southwestern US) and the 2009 pandemic (Southeastern US). In order to quantify the role of distance on spatial spread, we plot pairwise synchrony in epidemic onsets as a function of geographic distance (Fig 4). In all seasons except 2006/2007 and 2008/2009, pairwise synchrony decreased with distance up to 1500–2000 km and departed significantly from patterns expected under the null hypothesis of complete spatial randomness. This spatial signature was most pronounced in 2003/2004, 2005/2006 and the 2009 pandemic, in which between 30–58% of synchrony estimates were significantly different from the null (Fig 4). In 2003/2004 there was a trend towards increasing synchrony between US coasts, as indicated by similar epidemic timings in pairs of locations separated by 2500–3000 km compared to pairs at intermediate distances.

### Geographic origin of epidemics

**Fig 5:**
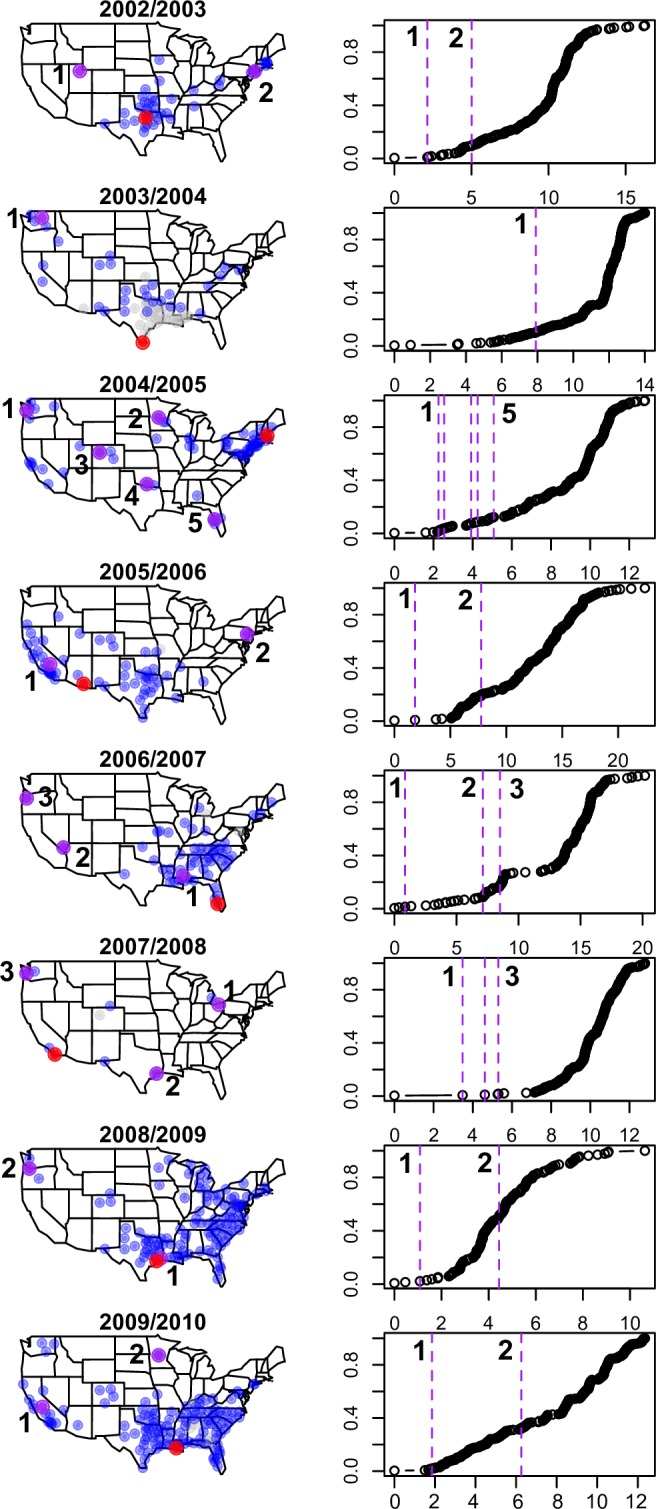
Long range transmission events and potential origins of each epidemic. For each season, the purple circles indicate long-range transmission events (i.e. locations far from the set of infectious locations that obtain influenza regardless). Blue circles are locations infected within a two-week window from the *last* long-range event. Red circles depict the potential outbreak origin in each epidemic. Grey circles are infected locations before the first long-range event occurred in each season. Panels on the right indicate when in the epidemic long-range events occurred. Note that the methods used to identify long-range transmission events and estimate the origin of each epidemic are agnostic of one another; in 2008/2009 an early “long-range transmission event” appears near the estimated origin of the epidemic.

To better identify the initial focus of each outbreak, we adapted a previously developed method [6], where the most likely source location is identified as the location that maximizes the correlation between onset times and the geographic distance to the potential source (see methods). Notably, of the eight seasons studied, seven were likely seeded in the Southern US (Fig 5 and supporting S1 Text, Figure A4). The relationship between influenza timing and distance to the source location was particularly pronounced in four seasons (2002/2003, 2003/2004, 2005/2006 seasons and the 2009 pandemic, supporting S1 Text, Figure A4), echoing earlier synchrony results (Fig 4). The distance signature generally subsided after 1000–1500 km, pointing to the importance of one or several long-distance transmission events effectively acting as new sources and obscuring further distance effects.

### Long-distance transmission events

We sought to quantify the number and locations of long-distance transmission events in each season, based on the minimum distance between a newly infected location and the set of previously infected locations. We generated an empirical distribution of this statistic across seasons that takes into account uneven geographic sampling, and defined events occurring in the upper 1^st^ percentile of this distribution as long-range transmission events (methods and supporting S1 Text, Figure A3). Between 1 and 5 such events were identified per season (Fig 5); ~55% of these events were associated with increased influenza activity in adjacent areas within a two-week window (within a radius of ~570km). Long-range events generally occurred early in the epidemic, within the first ~10–30% of cities infected (Fig 5, supporting S1 Text, Table A2).

### Mechanistic models of influenza spread

Next, we developed a mechanistic transmission model to identify essential drivers of the spatial process in each season, inspired by previous work on influenza [18,19] and the foot-and-mouth disease epidemic in the UK [22] (see methods). The model captures the directionality of the infectious process, allowing infected locations to transmit disease to susceptible ones. The probability of transmission is a function of the geographic distance between susceptible-infectious pairs (power-law parameter, γ), and each location’s susceptibility is treated as a function of its population size (power-law parameter *μ*), the number and position of nearby locations (normalization parameter, ε) and external seeding (*ρ*). We also considered two alternative formulations of this model, where geographic distance is replaced by one of two proxies of human mobility based on work commutes or air travel.

**Fig 6:**
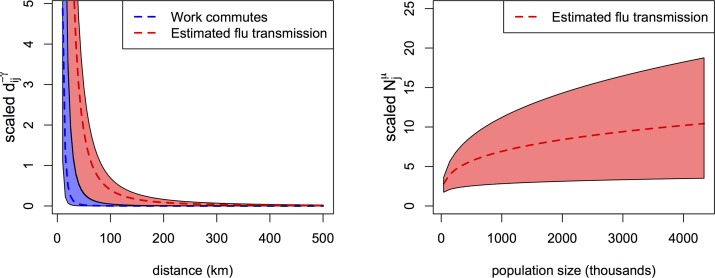
Spatial transmission kernels and demographic effects summarized across seasons. Left panel. Work commutes scale with geographic distance according to a power law of 3.3 (95% CI: 2.66 to 3.95); this relationship is depicted in blue (See also supporting S1 Text, Figure A6). Based on our distance-based transmission model, the force of infection between an infectious and susceptible city, *λ*_*ij*_, scales with geographic distance according to a median estimated power law of 2.2 (range: 2.08 to 2.65), depicted in red. This indicates that for a 10-fold decrease in distance between an infectious and susceptible pair of cities, the hazard of infection on the susceptible city increases by a factor of ~158 (range: 120–467). Right panel. The force of infection on a susceptible city, *λ*_*j*_, scales with its population size according to a median estimated power law of 0.28 (range: 0.15 to 0.35). This indicates that for a 10-fold increase in population size, the hazard of infection on the susceptible city increases by a factor of 1.9 (range: 1.4–2.2).

Overall, models using the geographic distance metric fit the data best across seasons (Table 2). The range of power-law distance exponents was narrow ($\hat{\gamma }$ = 2.1 to 2.7, Table 3), with a median value of 2.2, indicating a sharp decay in the risk of influenza infection between a susceptible-infectious pair of locations as the geographic distance between the pair increases (Fig 6). The most extreme values of the distance exponent ($\hat{\gamma }$>2.5), were found in the 2006/2007 season and the 2009 pandemic. In the 2003/2004 season, the sharp decline in the risk of transmission as a function of pairwise distance ($\hat{\gamma }$ = 2.2) indicates that the observed synchrony between the east and west coasts (Fig 4, Panel 2) is likely the result of radial spread from locations in the Southern and Midwestern US.

**Table 2:** Change in AIC values for the spatial models using geographic distance, work commutes and domestic air traffic as the links between locations driving inter-city spread, compared to the model with the minimum AIC in each season. The number of cities available for analysis is based on the subset of 310 cities for which onset time could be estimated and which could be successfully matched with county-level commute data from the census. Spatial models were not fit to the 2008/2009 epidemic because of lack of detectable influenza onset times in a sufficient number of locations.

Season	Cities (#)	Geographic distance	Work commutes	Air traffic
**2002/2003**	221	0	25.46	78.87
**2003/2004**	290	0	30.16	105.13
**2004/2005**	270	0	24.53	129.61
**2005/2006**	218	0	45.32	161.8
**2006/2007**	198	9.27	0	-*
**2007/2008**	270	0	8.31	59.81
**2009/2010**	281	0	57.42	247.09

*Unique maximum partial likelihood estimates not identified.

**Table 3:** Maximum partial likelihood parameter estimates from the model $\lambda \;(t_{j}|H_{t_{j}},\mu ,\gamma ,\varepsilon ,\rho )=\lambda_{0}(t_{j})[N_{j}^{\mu }\sum_{i\in I_{t_{j}}}\frac{d_{ij}^{\gamma }}{({\sum_{k:k\neq j}d_{jk}^{\gamma })}^{\varepsilon }}+\rho ]$, utilizing each of the three distance metrics considered (geographic distance, work commutes and air traffic). Spatial models were not fit to the 2008/2009 epidemic because of lack of detectable influenza epidemic onset times (estimated from outpatient influenza-like-illness time series) in a sufficient number of locations.

*Seasons*:	2002/2003	2003/2004	2004/2005	2005/2006	2006/2007	2007/2008	2009/2010
**Geographic Distance**							
$\hat{\gamma }$ (SD)*	2.08 (0.2)	2.22 (0.15)	2.30 (0.19)	2.19 (0.13)	2.65 (0.33)	2.18 (0.22)	2.64 (0.16)
$\hat{\mu }$ (SD)	0.11 (0.07)	0.21 (0.06)	0.35 (0.08)	0.31 (0.08)	0.33 (0.09)	0.28 (0.075)	0.15 (0.070)
$\hat{\varepsilon }$ (SD)	0.59 (0.09)	1.06 (0.07)	0.96 (0.07)	0.86 (0.09)	0.62 (0.12)	0.91 (0.07)	1.16 (0.05)
$\hat{\rho }$ (SD)	0.0014 (0.0012)	0.031 (0.048)	0.99 (0.47)	0 (1.3E-5)	0.084 (0.035)	0.36 (0.20)	0.28 (0.15)
**Work commutes**							
$\hat{\gamma }$ (SD)	0.60 (0.06)	0.67 (0.04)	0.56 (0.05)	0.81 (0.06)	0.73 (0.07)	0.63 (0.06)	0.71 (0.04)
$\hat{\mu }$ (SD)	-0.16 (0.12)	0.09 (0.10)	0.05 (0.11)	0.03 (0.11)	0.31 (0.09)	0.05 (0.12)	0.03 (0.10)
$\hat{\varepsilon }$ (SD)	0.74 (0.07)	0.9 (0.05)	0.74 (0.08)	0.76 (0.06)	0.69 (0.07)	0.7 (0.07)	0.93 (0.05)
$\hat{\rho }$ (SD)	0.0078 (0.005)	0.022 (0.016)	0.10 (0.07)	0.15 (0.07)	20.29 (9.47)	0.32 (0.21)	0.01 (0.008)
**Airline traffic**							
$\hat{\gamma }$ (SD)	2.25 (0.64)	1.54 (0.23)	1.41 (0.52)	0.70 (0.38)	-**	1.88 (0.52)	1.28 (0.34)
$\hat{\mu }$ (SD)	-0.09 (0.12)	0.14 (0.09)	0.32 (0.11)	0.29 (0.11)	-**	0.29 (0.11)	0.15 (0.11)
$\hat{\varepsilon }$ (SD)	0.98 (0.02)	0.99 (0.02)	0.98 (0.04)	1.04 (0.04)	-**	0.98 (0.02)	1.06 (0.02)
$\hat{\rho }$ (SD)	0.064 (0.024)	0.23 (0.073)	19.69 (7.05)	1.45 (0.67)	-**	6.69 (2.90)	0.20 (0.08)

*For ease of parameter interpretation, the model using the geographic distance metric was fit by replacing *γ* with – *γ* (see methods).

**Unique maximum partial likelihood estimates not identified.

Estimates of *μ*, the dependence of a location’s susceptibility on its population size ranged from ~0.1–0.35, with a median value of 0.28 (Table 3), indicating that more populated locations are at relatively higher risk for influenza transmission (Fig 6). Parameter estimates for external seeding, *ρ*, varied several orders of magnitude between seasons, from ~0 in the 2005/2006 season to 0.98 in the 2004/2005 season. External seeding was weaker in seasons with particularly radial patterns in epidemic onsets—2002/2003, 2003/2004, 2005/2006. The large heterogeneity in estimates of *ρ* across seasons highlights the variable nature of long-distance transmission events in seeding epidemics during the seasons studied.

Spatial models based on geographic-distance outperformed those integrating mobility-indices in all seasons except for 2006/2007, where the work commutes-based model fit marginally better (Table 2). Models incorporating work commutes systematically outperformed those using air traffic (Table 2, see also supporting S1 Text, Figure A5 for a comparison of connectivity between the two mobility proxies). Model estimates suggest that the risk of influenza transmission scales with work commutes according to a power law exponent between 0.6–0.8 (median = 0.67, Table 3). To better understand the differences between the models considered here, we explored the relationships between work commutes, air traffic and geographic distance (supporting S1 Text, Figure A6). We found that work commutes tend to be more localized than influenza transmission (distance power-laws of ~3.3 for work commutes and ~2.2 for influenza transmission, Fig 6), while air traffic did not scale with geographic distance (supporting S1 Text, Figure A6). Models relying on mobility proxies did not indicate a strong role for population size (*μ*) (Table 3).

Interestingly, the importance of the normalization parameter (*ϵ*) is seen across all models, as estimates were generally close to 1 and always excluded 0 (Table 3). This provides support for a density-independent infectious process, at least at the level of sampling in this dataset. For illustration, a map of the normalization effect indicates that a density-dependent transmission model (*ε* = 0) would tend to under-estimate influenza risk in cities with few close neighbors (supporting S1 Text, Figure A7). In other words, under the current sampling scheme, the least connected cities, generally located in the central part of the US, have a stronger risk of infection than their neighborhood alone would predict in the absence of normalization.

Finally, we also considered more complex models incorporating other demographic variables (population size of the infectious locations) and climatic information (absolute humidity in susceptible locations), but none of these factors provided substantial improvements to the model fit (supporting S1 Text, Table A3). Sensitivity analyses revealed that parameter estimates were robust to the inclusion of measurements errors in onset times (supporting S1 Text, Figure A13), and were comparable to those estimated under a fully-specified likelihood (instead of the partial likelihood used here) (supporting S1 Text, Table A4). These analyses also revealed that the risk of influenza infection can vary substantially over the time-course of an epidemic (supporting S1 Text, Table A5, Figures A8 and A9). In further sensitivity analyses, we refit the semi-parametric models constraining the highly-variable external seeding term *ρ* to 0. Under this nested model, estimates of the distance exponent *γ* were largest in the 2003/2004, 2005/2006 seasons and the 2009 pandemic, aligning with patterns of localized spread observed in maps of epidemic onsets (Fig 2) and synchrony analyses (Fig 4; supporting S1 Text, Table A8).

## Discussion

Informed disease control and pandemic planning rely on understanding the mechanisms of and variability in the spatial transmission of influenza. Here we studied the spatial and temporal dynamics of annual influenza epidemics in the US over eight seasons, leveraging uniquely spatially-resolved medical claims data on outpatient influenza-like-illnesses (ILI) through an active research collaboration with a data-warehouse company. To our knowledge, this is the most detailed and comprehensive study of the city-level spread of influenza over multiple seasons to date, revealing a number of insights and generating hypotheses about the mechanisms of disease transmission.

### Human mobility and the spatial spread of influenza

While it is well accepted that international air travel plays a crucial role in the global dissemination of seasonal and pandemic influenza viruses [3,6,7,11], the role of air traffic in the regional spread of influenza remains debated [1,12–14]. Our study points to a predominantly localized mode of influenza transmission within the continental US, even though the exact pathways of epidemic spread and initial seeding events were variable across seasons. The estimated risk of transmission decays sharply with geographic distance from an infected location according to a power-law of ~2.2 (Range: 2.1–2.7). A particularly localized, radial spatial structure was observed in the 2003/2004 and 2005/2006 epidemics dominated by A/H3N2 viruses and, as reported previously [19], in the fall wave of the 2009 pandemic dominated by A/H1N1pdm. We extended previous models of influenza transmission by explicitly connecting disease data with human mobility proxies and found that models driven by geographic distance or work commutes systematically outperformed those driven by air traffic. Taken together, these results do not support domestic air traffic as the dominant mode of spatial dissemination of influenza in the US.

We identified between one and five long-range transmission events per season, most occurring in the first third of each epidemic and half contributing successfully to the observed dynamics via secondary onward transmission. In model-based analyses, the estimated effect of external seeding varied more than 10-fold across seasons. Whether domestic or international air traffic is responsible for these long-range events and the initial seeding of the epidemic is an important area for future research, which would benefit from complementary analysis of phylogenetic data [5,11,23].

Previous work has indicated that work commutes are an important driver of the inter-state spread of influenza in the US [1,14]; here we show that at the scale of cities, models utilizing geographic distance outperformed those using work commutes. The range of connectivity implied by the work commute data may be too narrow to accurately capture influenza transmission, which occurs on a broader spatial scale, likely contributing to the better fit of distance-based spatial models (Fig 6). Further differences in model fit may stem from the substantially higher degree distribution of the geographic distance network compared to the between-city work-commute network, thereby allowing many more possible pathways for epidemic spread in distance-based models (see supporting S1 Text, Figure A5). Reassuringly, however, there was excellent internal consistency between parameter estimates for the work commute and distance-based models (see supporting S1 Text).

### Demography and the spatial spread of influenza

Prior work has indicated that influenza spread at the state-level in the US is dominated by hierarchical spread between populous states (perhaps driven by inter-state work commutes) [1], but this work was limited by low-resolution (state-level) mortality data and patterns were averaged across several decades. Our model-based analysis of higher resolution outpatient ILI visits indicates that while more populated locations are at relatively higher risk for influenza transmission compared to less populous locations, this effect is not strong enough for hierarchical spread to predominate over local spread. Indeed, a susceptible city’s population size contributed only marginally to its risk of obtaining influenza early in an epidemic. It is important to note that this estimated relationship may be confounded by differences in the age-structures of large and small populations, which are not accounted for in our model. In a supplementary analysis (supporting S1 Text, Figure A14) we demonstrate that more populous counties are enriched with persons between 20 and 50 years of age, a segment of the population with presumably higher rates of travel (both local and long-distance). Enrichment for this segment of the population in larger counties may confound the relationship between the risk of influenza infection and population size.

In complementary analyses, we estimated spatial synchrony in ILI data using the approach proposed in Viboud et al. [1], both at the original resolution of cities and with data aggregated at the state-level. This sensitivity analysis suggests that the effects of workflows and demography are more pronounced at the state-level than at the city-level, validating earlier results [1] (supporting S1 Text, Table A6, Figures A10, A11). In contrast, the relationship between synchrony and geographic distance predominates at the scale of cities, indicating that spatial data aggregation likely biases the precise characterization of disease spread. The availability of finely spatially-resolved disease data is therefore essential to accurately capture the mechanisms of disease dissemination [24].

### Absolute humidity and the spatial spread of influenza

In contrast to previous work [15,16], our analysis did not find that absolute humidity was an important driver of the spatial spread of influenza. Absolute humidity did not affect the susceptibility of individual cities to influenza infection (supporting S1 Text, Table A3), though our models did not allow for spatial variation in the effect of absolute humidity. This finding is consistent with prior studies of the 2009 pandemic in the US [19] and Europe [25]. Furthermore, our analysis suggests seven of the eight epidemics studied likely originated in the Southern US, several in humid areas of Texas and Louisiana, a pattern that does not fit with the concept that low humidity favors influenza transmission [15]. Analyses of longer epidemiological records should shed light on whether the preferred Southern origin of epidemics is due to frequent seeding of influenza in the Southern US (potentially from Central and South America), or if demographic or geographic conditions favor early influenza activity in the South. Importantly, our study did not address the potential role of environmental factors on the local dynamics of influenza, particularly in the weeks immediately following the estimated onset times in each city.

### Schools and the spatial spread of influenza

Prior work on the 2009 pandemic has indicated a role for school openings in modulating the spatial structure of influenza spread during that season [19,26]. This was especially important in 2009 because (1) influenza transmission in the US occurred much earlier in the year (August-October) than for seasonal outbreaks, coinciding with the start of the fall school term, and (2) there is substantial geographic variation across start dates for US school fall terms, with earliest school openings in the Southeastern US. In contrast, seasonal influenza outbreaks tend to occur later in the year (onset dates Oct-Jan), typically well into the school fall term. While it is possible that the Christmas holiday break may modulate spatiotemporal patterns in seasonal influenza spread, the timing of this break is more uniform across the US and is not as likely to contribute to spatial variation in spread.

### Effect of antigenic novelty on spatial spread of influenza

Given the great interest in predicting how a novel influenza virus will spread through a largely susceptible population, two high transmission seasons merit special attention: the 2003/2004 drift-variant A/H3N2 epidemic and the fall wave of the 2009 pandemic (previously studied in [19]). Yang et al. [20] showed that the pre-season population-level susceptibility (~70%), effective reproductive number (1.4–1.6), and overall attack rates (24–33%) were similar for these two major influenza seasons, and in the higher range of seasonal influenza epidemics. We found that in both seasons, influenza virus circulation originated in the Southern US and began earlier in the year than is typical. The relatively slow spread (9–12 weeks) and strong spatial signature of these two seasons is surprising because theory suggests that more transmissible influenza viruses, associated with higher effective reproduction numbers, should spread faster and less radially (because long-distance introductions are more likely) than less transmissible viruses [1,19]. The net spatial effect of increased transmissibility is likely modulated by the mobility patterns of the most susceptible hosts, which may vary substantially with age and across seasons.

Though a satisfying explanation for these findings remains elusive, the spatial diffusion of novel influenza viruses appears more localized than previously speculated and multiple introductions of influenza may be required to seed an epidemic in any given city, as the probability that a single introduction sparks an epidemic is low. Because commuting flows are on average one order of magnitude larger than air traffic volume [13], predominantly localized transmission is consistent with the idea that local connectivity (which includes work commutes) is the main driver of epidemic spread [1]. Finally, this finding may also highlight the importance of children in spreading influenza over short distances, an age group that also tends to be disproportionately impacted during times of novel influenza virus circulation [27,28].

We have not considered the role of human behavior change in shaping the spatiotemporal dynamics of epidemics [29], but we anticipate that the perception of risk during seasonal influenza epidemics is relatively consistent across seasons, and suspect this may play only a minor role in modulating spatial spread. During the 2009 pandemic, the US experienced a widely publicized “herald wave” of H1N1pdm transmission during the spring. By the fall, the US population was broadly aware that H1N1pdm infection was generally mild, likely reducing reactive behavior change during the fall wave of the pandemic. In our study, we model influenza epidemic onset times, rather than the full epidemic dynamics in each location, and is thus our approach is less sensitive to reactive behavior changes in response to growing case counts near the peak of the outbreak.

### Modeling strategies and future developments

Numerous strategies exist for modeling epidemic spread. Inspired by previous work on the foot-and mouth-disease epidemic in the UK [22], we employed semi-parametric transmission models to quantify the risk of infection as a function of demographic factors and different distance metrics across several seasons. Previous studies have used fully parametric models and have focused on a single epidemic with the goal of capturing the entire epidemic process [19,30–33]. We found that fully parametric models often require time-varying baseline hazards to capture the temporal dynamics of influenza epidemics (supporting S1 Text, Table A5, Figures A8 and A9). Accordingly, a constant baseline hazard was unable to fully capture the temporal dynamics of the 1918 epidemic, with model-predicted onsets times occurring earlier on average than observations [18]. For the 2009 pandemic, information on school openings was necessary to allow the baseline hazard for transmission to change over time (and space) [19]. In this context, the partial likelihood method [22] provides clear advantages in obtaining robust estimates for key spatial covariates while obviating the need to specify the form of the time-varying baseline hazard. In sensitivity analyses, we found that parameter estimates for the spatial component of the model were robust to the choice of baseline hazard in fully-parametric model formulations (supporting S1 Text, Table A4). One disadvantage of the semi-parametric method, however, is that it does not allow simulations of epidemic trajectories over time and cannot be applied for real-time predictions, as the baseline hazard is not fully specified.

Our study is subject to several limitations. First, and most importantly, our study was limited to eight seasons, making our conclusions difficult to generalize broadly given year-to-year variability in circulating influenza strains. Second, although ~80% of the US population was eventually captured in this dataset, this percentage was lower in earlier years of data collection, and the Midwestern US was geographically under-sampled. Third, our analysis relies critically on the accurate estimation of epidemic onsets in each location, which is particularly challenging during mild epidemics because the influenza signal is weaker. To address this issue, we (1) analyzed data only from locations with sufficiently robust influenza signals, (2) quantified the uncertainty in epidemic onset estimates, and (3) computed onset times using more “traditional” methods (sinusoidal regression models), which showed excellent agreement with our estimates (supporting S1 Text, Figure A12). Further, we have shown that the main results of our study are robust to moderate uncertainties in the epidemic onset times (supporting S1 Text, Figure A13). Fourth, our dataset is based on ILI records rather than laboratory-confirmed influenza cases. Though outpatient ILI captures the aggregate effects of a number of respiratory pathogens, previous work has shown strong agreement with laboratory-based indicators of influenza virus activity at the regional and city level [21]. Furthermore, our analysis is focused on influenza epidemic onset times, which are identified using a linear-spline method separating background ILI rates from rapid case growth due to influenza activity. This approach controls for the effect of other respiratory pathogens in the peri-epidemic period.

Another caveat relates to the co-circulation of several influenza virus subtypes or strains in some winters. Syndromic (ILI) time series capture the aggregate effects of these viruses, making it challenging to disentangle the transmission dynamics of one virus from another. We speculate, however, that co-circulation would superpose multiple spatial patterns thereby biasing analyses towards spatial randomness; reassuringly, we observed a spatial signature during epidemics in which multiple subtypes co-circulated (2002/2003, 2004/2005, and 2005/2006). Importantly, this caveat does not apply to the severe and radially diffusive influenza seasons in 2003/2004 and 2009, which were marked by overwhelming predominance of a single antigenically-novel strain.

We relied on gravity-type power-law models of influenza transmission and incorporated empirical data on human mobility; these models have been validated as parsimonious approximations of both human movement [1,34] and influenza spread [13,18,19]. A major limitation of these models, however, is that they consider the pairwise interaction between infectious/susceptible cities, but ignore higher order interactions, which may prove to be essential in capturing realistic patterns of human movement. In reality, some combination of work commutes, air travel and other local movements likely drive disease dissemination, and ideally these different connectivity metrics should be integrated in a single model rather than analyzed separately. Because the observed mobility networks are captured on different scales both geographically and temporally, it is not clear how best to combine information across such different networks [35,36]. Finally, the observed data represent a sample of locations within the network of interacting cities; the effects of geographic sampling on parameter estimation is poorly understood. Further work is needed to develop realistic model formulations that connect multiple mobility indices and disease datasets at appropriate temporal and spatial scales.

In line with previous work on the spatial spread of influenza [19] our analysis focuses on epidemic establishment in each location under study, rather than the time of influenza introduction. Epidemic establishment is more complex than the mere importation of influenza cases, as it requires sustained chains of local transmission, which may depend on climatic or virus-specific factors, among others. In our study, the unit of analysis is the city, and only after establishment of sustained local transmission in each city are there sufficient numbers of cases to allow for influenza spread to other cities, as built into the model framework utilized here. The frequency of viral introductions and specific viral migration pathways would be interesting to study but are best addressed by phylogeographic analyses, assuming appropriate sampling of viral sequences early in the epidemic [5].

### Conclusions

Our analysis of the spatiotemporal dynamics of influenza across eight seasons using granular surveillance data provides several new insights into how geography, human mobility and immunity intermesh to shape the dissemination of influenza in the US. Spatial dissemination is radial and localized, with domestic air traffic playing little role. Intriguingly, epidemics have a propensity to begin in the Southern US, a finding contradictory to existing concepts of environmental forcing on influenza transmission [15]. Two seasons marked by the circulation of novel viruses (2003/2004 epidemic and the 2009 pandemic) were unique in that influenza arrived earlier in the year than is typical and patterns of spread were particularly localized and radial. Novel influenza viruses appear to spread more locally than previously speculated, perhaps driven by a younger mean age at infection and in turn a decrease in the range of mobility of susceptible hosts. Most importantly perhaps, our work points to a need for highly granular epidemiological datasets to deepen our understanding of influenza transmission, beyond the data resolution available from traditional surveillance schemes. Moving forward, validation of our findings using longer epidemiological records and pathogen genetic information at the same spatial scale will be essential [5]. Further work should also concentrate on the importance of data resolution, antigenic novelty, and age-specific differences in prior immunity on spatial transmission.

## Materials and methods

### Epidemiological data

We compiled weekly time series of influenza-like-illnesses (ILI) from 2002–2010 under a collaborative research agreement with IMS Health, a private data and analytics company that collects de-identified CMS-1500 electronic medical claims from outpatient physician visits throughout the US. The system covered 61.5% of US physicians in 2009 [21]. ILI records included all visits with a direct mention of influenza, or fever combined with a respiratory symptom, or febrile viral illness (ICD-9 487–488 OR [780.6 and (462 or 786.2)] OR 079.99) [21]. Data were stratified by 3-digit zip codes of physicians’ offices and aggregated further according to sectional center facilities as defined by the United States Postal Service, a division akin to a city or occasionally a county. After exclusion of locations with fewer than 100,000 inhabitants to decrease demographic noise, and matching with census population data for denomination, we obtained stable ILI records for 310 locations, which we thereafter denote as ‘cities’ for simplicity. Previous work has shown that standardized ILI time series derived from this surveillance system accurately capture influenza virus activity on a regional and local basis, where standardization is obtained by taking the ratio of weekly ILI visits to the total number of physician visits that week in a given locale, per 100,000 population [21]. Further, these ILI time series are appropriate for spatial modeling [19].

### Human mobility proxies

#### Work commutes

We obtained data on county-to-county work commutes from the 2000 US Census. Data reflect responses to the question of where a person spent the most time working in the past week and where said person lives. Therefore, these data contain information on occasional work-related trips as well as on journeys to an individual’s typical workplace [37].

#### Domestic air travel

For each influenza season, we obtained domestic air travel data from the first month with substantial influenza transmission, as provided online from the US Department of Transportation (www.transtats.bts.gov). We used the “T-100 Market Airline Traffic Data”, which for each month of a given year, contains information on the number of enplaned passengers travelling between any two US airports on direct flights. Passengers changing planes at an intermediate destination airport would be counted twice, once between the initial airport and the intermediate airport, and once between the intermediate airport and the final destination.

### Epidemic onset determination

Our goal was to estimate the onset time of influenza in each location for each of the 8 available seasons, 2002/2003-2009/2010. First we selected locations with a sufficient rise in ILI above baseline for further study. Specifically, for each location and season, we computed the difference between the maximum ILI incidence and the maximum ILI incidence in the first 10 weeks of the season (before influenza circulation). We generated an empirical distribution of this statistic across seasons and locations, and excluded locations if their observed statistic fell in the bottom 20^th^ percentile of this distribution. This produced between 135–306 locations per season, based on the strength of the ILI signal in each location.

Among these selected locations, we considered weekly time series of ILI in each season from the first week of June to the first week with the maximum incidence of ILI. For each season and location separately, we fit piecewise linear models to capture the timing of the change-point in incidence which corresponds to the epidemic onset:

$$Y_{j,t}=\beta_{0,j}+\beta_{1,j}t+\beta_{2,j}{\left(t-t_{j}\right)}^{+}+\epsilon_{j,t}$$

$$\epsilon_{j,t}∼N(0,\vartheta_{j}^{2})$$

In the above, (.)^+^ denotes the positive portion of its argument, *j* indexes the location and *t* indexes the week, so that *Y*_*j*,*t*_ is the weekly standardized ILI incidence. ε_j,t_ is the model error term, assumed to be normally distributed with zero-mean and variance θ_j_^2^. The knot-location of the linear spline term, *t*_*j*_, represents the epidemic onset time at location *j*. Finding the optimal knot location is often a difficult problem, primarily because there are often many local optima in the surface of the objective function [38]. We proceeded as follows. Let *L*(*β*_0,*j*_, *β*_1,*j*_, *β*_2,*j*_, *t*_*j*_) be the likelihood function of the parameters given the data, *Y*_*j*,*t*_. Define the profile likelihood function $L_{1}(t_{j})={max}_{\beta_{0,j},\beta_{1,j},\beta_{2,j}}\;L(\beta_{0,j},\beta_{1,j},\beta_{2,j},t_{j})$. In other words, for a fixed value of *t*_*j*_, *L*_1_(*t*_*j*_) is the likelihood maximized over the other parameters. To estimate the epidemic onset time *t*_*j*_, we maximized log*L*_1_(*t*_*j*_) using a Nelder-Mead simplex algorithm. We computed the inverse of the second derivative of $logL_{1}(\hat{t_{j}})$ as an estimate of the variance of the estimated onset time $\hat{t_{j}}$, denoted $\hat{\sigma_{j}^{2}}$. This procedure was repeated independently for each location and season and is illustrated in supporting S1 Text, Figure A1.

All analyses in our paper focus on the estimated influenza epidemic onset time in each location, $\hat{t_{j}}$. Conceptually, this time represents epidemic establishment in each city, rather than the time of influenza arrival/importation. Epidemic establishment is epidemiologically interesting because it requires sustained chains of transmission to take hold, which may depend on climatic or virus-specific factors. Influenza arrival or importation, on the other hand, simply represents the first case of influenza in a given location, irrespective of whether this introduction sparks sustained local transmission and a subsequent epidemic.

### Descriptive analyses

#### Spatial synchrony of epidemics

For each season separately, we computed the difference (in absolute value) in estimated timing of influenza onset between each pair of locations and the geographic distance separating them. We created bins of 50 kilometers in length ((0–50), [50–100), [100–150)…[4350–4400)) and for each bin computed the mean of the pairwise difference in epidemic onset times. We used a jackknife procedure to obtain variance estimates for the estimated mean in each bin and used these estimates to obtain a pointwise 95% confidence interval for the mean in each bin.

To compare against complete spatial randomness in each season, we generated 150 artificial datasets by permuting the original onset times in each location and computed the mean and standard deviation of the absolute value of the difference in epidemic timing in each bin. To obtain summary statistics of the strength of the spatial signature of each epidemic, we tabulated the proportion of bins significantly different from the null of complete spatial randomness.

#### Identifying long-distance transmission events

Long-range transmission events represent an important aspect of infectious disease dynamics. Here we propose a simple method of identifying such events using estimates of influenza epidemic onset times in each location and season, $\hat{t_{j,s}}$.

The method is based on identifying outliers in the distribution of pairwise distances between newly infected cities and the set of infectious cities at the previous time step. Let *C* be the set of locations in the network. At time *t*_*j*_, we can divide *C* into the set of infectious locations (that can transmit infection) $I_{t_{j}}$, and the set of susceptible locations $S_{t_{j}}$ (that can become infected; the risk-set):

$$I_{t_{j}}=\{k:t_{k}<t_{j}\}$$

$$S_{t_{j}}=\{k:t_{k}\geq t_{j}\}$$

For location *j*, we can compute the minimum distance between location *j* and infectious cities in $I_{t_{j}}$ as the most likely pathway of infection in a distance-driven spatial transmission process:

$$d_{j}=\underset{k}{\min}d_{jk}$$

for $k\in I_{t_{j}}$. Because we consider only a finite set of locations, we must take into account the minimum distance between city *j* and any other location in the network:

$$D_{j}=\underset{i}{\min}d_{ji}$$

for *i* ∈ *C*. Under the hypothesis of a purely spatial process, we would expect locations to be infected by a close neighbor, which we approximate as the distance to the nearest neighbor, implying *d*_*j*_ − *D*_*j*_ ≈ 0. For each city in each season, we computed *d*_*j*_ − *D*_*j*_, and defined long-range transmission events as infections in those locations falling in the 99^th^ percentile of the distribution of *d*_*j*_ − *D*_*j*_ across all seasons under study. The choice of threshold is by nature arbitrary; the 99^th^ percentile identifies jumps that lie safely in the tail of the *d*_*j*_ − *D*_*j*_ distribution (supporting S1 Text, Figure A3).

#### Identifying potential sources of an outbreak

To identify the geographic origin of the epidemic in each season, we adapted the “effective distance” method of Brockmann *et al*. [6], which relies on the concept that epidemics spread radially from an origin, so that epidemic onset times are linearly related to effective distance from the source. This method can be used to (i) identify the correct distance metrics for the disease system under study (effective distances can be based, for example, on geographic distance or mobility proxies) and (ii) identify the most likely origin of the outbreak. Here we focus on the second aspect, and systematically test a large set of locations as potential sources.

Due to error in our epidemic onset estimation procedure and reporting error, we considered that any one of the first 10% of locations infected in a season could be a potential source for the epidemic. For each potential source location, we computed Pearson’s correlation coefficients for the relationship between each location’s influenza onset time and its geographic distance to the source. We selected the “most likely” origin as the location with the maximum value of the correlation coefficient (supporting S1 Text, Figure A4).

#### Semi-parametric mechanistic model of the infectious process

The goal of this study was to model the spatial risk of influenza transmission in each of the eight seasons for which robust data were available. In each season we treated the finite set of locations under study as linked populations (the strength of the connections between locations is based on the distance between them) in which influenza can be transmitted from an infected population to a susceptible one. We adapt the partial likelihood approach for spatiotemporal point processes originally proposed by Diggle for the foot and mouth disease epidemic in the UK [22,39]. Let *T*_*j*_ be a random variable representing the epidemic onset time of influenza in city *j* (*j* = 1…*N*_*s*_, *s* = 1…8 seasons). Let $H_{t_{j}}$ be the history of all past events prior to *t*_*j*_, and $\lambda (t|H_{t})$ be the conditional intensity for an event at time *t*, given $H_{t}$. Informally, the conditional intensity function is the rate at which events are expected to occur around time *t* given the history of the process prior to time *t*. A partial likelihood can be obtained by conditioning on the observed epidemic onset times *t*_*j*_ and considering the resulting likelihood for the time ordering of the events 1…N_s_. Adopting the notation of Diggle [22], the partial likelihood for city *j* is given by:

$$P_{j}=\frac{\lambda (t_{j}|H_{t_{j}})}{\sum_{k:t_{k}\geq t_{j}}\lambda (t_{k}|H_{t_{k}})}$$

Notice that the sum in the denominator is over the risk-set at time *t*_*j*_, the set of susceptible populations at time *t*_*j*_. Therefore *P*_*j*_ is the probability that city *j* is infected at time *t*_*j*_, as opposed to all other cities in the risk-set at time *t*_*j*_. The partial log-likelihood for all the data is given by:

$$L_{p}=\sum_{j=1}^{N_{s}}log\;P_{j}$$

In order to evaluate the partial likelihood, we assumed a semi-parametric model for the conditional intensity, which decomposes the hazard of infection in city *j* based on the proximity of city *j* to cities infected prior to *t*_*j*_:

$$\lambda (t_{j}|H_{t_{j}},\mu ,\gamma ,\varepsilon ,\rho )=\lambda_{0}(t_{j})[N_{j}^{\mu }\sum_{i\in I_{t_{j}}}\frac{d_{ij}^{\gamma }}{({\sum_{k:k\neq j}d_{jk}^{\gamma })}^{\varepsilon }}+\rho ]$$

Above, $I_{t_{j}}$ is the set of infectious cities at time *t*_*j*_, *N*_*j*_ is the population size of location *j*, *d*_*ij*_ is the geographic distance between locations *i* and *j*, and *λ*_0_(*t*_*j*_), the baseline hazard at time *t*_*j*_, is an infinite-dimensional nuisance parameter. Notice that this model has the same functional form as the spatial transmission models considered in Eggo et al. [18] and Gog et al. [19], with the added benefit that the time-varying baseline hazard, *λ*_0_(*t*_*j*_), need not be estimated to obtain estimates of parameters of interest – *μ*, *γ*, *ε*, *ρ*. The parameter *μ* captures how susceptibility to influenza infection varies as a function of population size. Large positive values of *μ* provide evidence for hierarchical transmission, suggesting that influenza preferentially will spread to larger population centers than smaller ones (within a stratum of distance). The power-law parameter *γ* captures how the risk of infection in a susceptible city varies as a function of distance to the set of infectious cities. This portion of the model incorporates the directionality of influenza transmission from an infected city to a susceptible one. We considered three different possibilities for *d*_*ij*_: (i) geographic distance (km), (ii) the number of county-to-county work commutes, and (iii) the number of domestic airline passengers travelling between each pair of locations. To allow for ease of interpretation of *γ*, when considering geographic distance for *d*_*ij*_, we fit the following model, in which *γ* is replaced by −*γ*:

$$\lambda (t_{j}|H_{t_{j}},\mu ,\gamma ,\varepsilon ,\rho )=\lambda_{0}(t_{j})[N_{j}^{\mu }\sum_{i\in I_{t_{j}}}\frac{d_{ij}^{-\gamma }}{({\sum_{k:k\neq j}d_{jk}^{-\gamma })}^{\varepsilon }}+\rho ]$$

Thus, highly positive values of *γ* indicate that the risk of infection decays sharply as a function of geographic distance between the infectious-susceptible pair. If the *d*_*ij*_ term in the model refers to the number of work commutes or airline passengers, based on the original model formulation, large positive values of *γ* indicate that the risk of infection increases as a function of the number of flows between the infectious-susceptible pair. The parameter *ε* captures how the connectivity of a city may vary as a function of the number and location of neighboring cities. The trivial case of *ε* = 0 corresponds to a density-dependent transmission model, in which the total infectious pressure on city *j* is the sum (weighted by distance and population size) of the contributions of each infectious city’s pressure on city *j*. In contrast, *ε* = 1 corresponds to a density-independent transmission model, indicating that the total infectious pressure on city *j* does not scale with the *number or distance* of neighbors, but rather the distance-weighted fraction of neighbors infected [18,19]. The parameter *ρ* represents the additive contribution of external seeding to the risk of influenza transmission in a susceptible location.

Because mobility data was available at the county-level while the epidemiological data were aggregated at the level of cities, we first matched each SCF with the county to which it belongs based on FIPS census information. The vast majority of cities matched to a single county, but 14 cities matched to 6 counties. In this case, we obtained a single epidemic onset for the six counties by taking the inverse-variance weighted average of epidemic onsets in the cities matched to those counties. To allow for comparisons across the three different spatial models, we analyzed data on the same network of cities for each season, and computed Aikaike’s Information Criterion (AIC) based on the maximized partial likelihood for each model (Table 2).

To construct a domestic airline traffic network that connects pairs of locations in our dataset, for each location we identified all airports within 100 km (or the nearest airport in the event it was beyond 100 km), and assigned all domestic traffic between pairs of airports. Both the work commute and air traffic networks were symmetrized so that flows from city *i* to *j* equal those from *j* to *i*.

#### Model estimation and computing

We maximized the partial log-likelihood over parameters *μ*, *γ*, *ε*, *ρ*, using a Nelder-Mead simplex algorithm as implemented in the general-purpose optimization function optim() in R v. 3.1.1. To obtain the asymptotic variance-covariance matrix associated with parameter estimates, we inverted the Hessian of the log-likelihood function evaluated at the maximum partial-likelihood estimates (we also verified that the Hessian was negative definite). For speed, the partial likelihood function was written in C++ and compiled in R v. 3.1.1 using the Rcpp package. Code and a simulated dataset are available upon request.

### Sensitivity analyses

#### Comparison of epidemic onset times with traditional methods

The results described in this paper rely on accurate estimates of influenza onset times in each location and season. We compared estimates of influenza onset times derived from our approach with traditional approaches based on harmonic regression models, and found excellent agreement (supporting S1 Text, Figure A12).

#### Incorporation of uncertainty in estimates of epidemic onset

The spatial model described above operates under the assumption that the onset times of influenza in each city are observed, though in reality, they are estimated from weekly time series of influenza-like-illnesses. So, along with each estimate of onset time in city *j* ($\hat{t_{j}}$), we also estimated the variance of the onset time in city *j (*$\hat{\sigma_{j}^{2}}$). To evaluate the impact of this uncertainty on parameter estimates from the model, for each city we drew the influenza onset time from a normal distribution centered at $\hat{t_{j}}$, with variance $\hat{\sigma_{j}^{2}}$. We generated 500 such datasets for each season and re-estimated the model described above, with comparable results. Histograms of parameter estimates from this procedure are provided in supporting S1 Text, Figure A13.

#### Parameter estimates in the setting of spatial randomness

To explore the range of parameter estimates in the setting of complete spatial randomness, we generated 500 artificial datasets by permuting influenza onset times between locations in a single season (2003/2004 epidemic). For each dataset, we re-estimated the model. Histograms and summary statistics for each parameter are provided in supporting S1 Text, Table A7.

#### Incorporating other demographic/environmental variables into the semi-parametric model

In addition to the simple four-parameter mechanistic transmission model introduced above, we considered also the potential role of other demographic and climatic variables in explaining observed patters in the timing of influenza epidemics. Specifically, for each season separately, we considered the six-parameter model:

$$\lambda (t_{j}|H_{t_{j}},\mu ,\phi ,\upsilon ,\gamma ,\varepsilon ,\rho )=\lambda_{0}(t_{j})[N_{j}^{\mu }{{AH}_{j}(t_{j})}^{\phi }\sum_{i\in I_{t_{j}}}\frac{N_{i}^{\upsilon }d_{ij}^{\gamma }}{({\sum_{k:k\neq j}N_{i}^{\upsilon }d_{jk}^{\gamma })}^{\varepsilon }}+\rho ]$$

All parameters are as before, with the addition of *AH*_*j*_*(t*_*j*_*)*, which measures absolute humidity in city *j* around the time of infection, *t*_*j*_. Here we considered the average absolute humidity for the two weeks before the time of infection. This expanded model also allowed for the “infectiousness” of cities $i\in I_{t_{j}}$ to vary by their population size *N*_*i*_ to some power. Parameter estimates and 95% confidence intervals from this model are provided in supporting S1 Text, Table A3.

## Supporting information

S1 Text Sensitivity analyses and supporting figures and tables. (PDF)

## References

[1] Viboud C, Bjørnstad ON, Smith DL, Simonsen L, Miller MA, Grenfell BT. Synchrony, waves, and spatial hierarchies in the spread of influenza. Science. 2006;312: 447–451. 10.1126/science.1125237 16574822

[2] Nelson MI, Simonsen L, Viboud C, Miller MA, Taylor J, George KS, et al Stochastic Processes Are Key Determinants of Short-Term Evolution in Influenza A Virus. PLOS Pathog. 2006;2: e125 10.1371/journal.ppat.0020125 17140286 PMC1665651

[3] Russell CA, Jones TC, Barr IG, Cox NJ, Garten RJ, Gregory V, et al The global circulation of seasonal influenza A (H3N2) viruses. Science. 2008;320: 340–346. 10.1126/science.1154137 18420927

[4] Bedford T, Riley S, Barr IG, Broor S, Chadha M, Cox NJ, et al Global circulation patterns of seasonal influenza viruses vary with antigenic drift. Nature. 2015;523: 217–220. 10.1038/nature14460 26053121 PMC4499780

[5] Viboud C, Nelson MI, Tan Y, Holmes EC. Contrasting the epidemiological and evolutionary dynamics of influenza spatial transmission. Philos Trans R Soc Lond B Biol Sci. 2013;368: 20120199 10.1098/rstb.2012.0199 23382422 PMC3678324

[6] Brockmann D, Helbing D. The hidden geometry of complex, network-driven contagion phenomena. Science. 2013;342: 1337–1342. 10.1126/science.1245200 24337289

[7] Grais RF, Ellis JH, Glass GE. Assessing the impact of airline travel on the geographic spread of pandemic influenza. Eur J Epidemiol. 2003;18: 1065–1072. 14620941 10.1023/a:1026140019146

[8] Tizzoni M, Bajardi P, Poletto C, Ramasco JJ, Balcan D, Gonçalves B, et al Real-time numerical forecast of global epidemic spreading: case study of 2009 A/H1N1pdm. BMC Med. 2012;10: 165 10.1186/1741-7015-10-165 23237460 PMC3585792

[9] Kenah E, Chao DL, Matrajt L, Halloran ME, Longini IM. The global transmission and control of influenza. PloS One. 2011;6: e19515 10.1371/journal.pone.0019515 21573121 PMC3089626

[10] Rvachev L A., Longini IM Jr.. A mathematical model for the global spread of influenza. Math Biosci. 1985;75: 3–22.

[11] Lemey P, Rambaut A, Bedford T, Faria N, Bielejec F, Baele G, et al Unifying viral genetics and human transportation data to predict the global transmission dynamics of human influenza H3N2. PLoS Pathog. 2014;10: e1003932 10.1371/journal.ppat.1003932 24586153 PMC3930559

[12] Brownstein JS, Wolfe CJ, Mandl KD. Empirical evidence for the effect of airline travel on inter-regional influenza spread in the United States. PLoS Med. 2006;3: e401 10.1371/journal.pmed.0030401 16968115 PMC1564183

[13] Balcan D, Colizza V, Gonçalves B, Hu H, Ramasco JJ, Vespignani A. Multiscale mobility networks and the spatial spreading of infectious diseases. Proc Natl Acad Sci U S A. 2009;106: 21484–21489. 10.1073/pnas.0906910106 20018697 PMC2793313

[14] Bozick BA, Real LA. The Role of Human Transportation Networks in Mediating the Genetic Structure of Seasonal Influenza in the United States. PLOS Pathog. 2015;11: e1004898 10.1371/journal.ppat.1004898 26086273 PMC4472840

[15] Shaman J, Pitzer VE, Viboud C, Grenfell BT, Lipsitch M. Absolute humidity and the seasonal onset of influenza in the continental United States. PLoS Biol. 2010;8: e1000316 10.1371/journal.pbio.1000316 20186267 PMC2826374

[16] Shaman J, Kohn M. Absolute humidity modulates influenza survival, transmission, and seasonality. Proc Natl Acad Sci. 2009;106: 3243–3248. 10.1073/pnas.0806852106 19204283 PMC2651255

[17] Haynes K, Fotheringham S. Gravity and spatial interaction models. Beverly Hills: Sage Publications; 1984.

[18] Eggo RM, Cauchemez S, Ferguson NM. Spatial dynamics of the 1918 influenza pandemic in England, Wales and the United States. J R Soc Interface R Soc. 2011;8: 233–243. 10.1098/rsif.2010.0216 PMC3033019 20573630

[19] Gog JR, Ballesteros S, Viboud C, Simonsen L, Bjornstad ON, Shaman J, et al Spatial Transmission of 2009 Pandemic Influenza in the US. PLoS Comput Biol. 2014;10: e1003635 10.1371/journal.pcbi.1003635 24921923 PMC4055284

[20] Yang W, Lipsitch M, Shaman J. Inference of seasonal and pandemic influenza transmission dynamics. Proc Natl Acad Sci. 2015;112: 2723–2728. 10.1073/pnas.1415012112 25730851 PMC4352784

[21] Viboud C, Charu V, Olson D, Ballesteros S, Gog J, Khan F, et al Demonstrating the use of high-volume electronic medical claims data to monitor local and regional influenza activity in the US. PloS One. 2014;9: e102429 10.1371/journal.pone.0102429 25072598 PMC4114744

[22] Diggle PJ. Spatio-temporal point processes, partial likelihood, foot and mouth disease. Stat Methods Med Res. 2006;15: 325–336. 16886734 10.1191/0962280206sm454oa

[23] Nelson MI, Njouom R, Viboud C, Niang MND, Kadjo H, Ampofo W, et al Multiyear persistence of 2 pandemic A/H1N1 influenza virus lineages in West Africa. J Infect Dis. 2014;210: 121–125. 10.1093/infdis/jiu047 24446525 PMC4162001

[24] Stark JH, Cummings DAT, Ermentrout B, Ostroff S, Sharma R, Stebbins S, et al Local Variations in Spatial Synchrony of Influenza Epidemics. PLOS ONE. 2012;7: e43528 10.1371/journal.pone.0043528 22916274 PMC3420894

[25] Merler S, Ajelli M, Pugliese A, Ferguson NM. Determinants of the Spatiotemporal Dynamics of the 2009 H1N1 Pandemic in Europe: Implications for Real-Time Modelling. PLOS Comput Biol. 2011;7: e1002205 10.1371/journal.pcbi.1002205 21980281 PMC3182874

[26] Tamerius J, Viboud C, Shaman J, Chowell G. Impact of School Cycles and Environmental Forcing on the Timing of Pandemic Influenza Activity in Mexican States, May-December 2009. PLoS Comput Biol. 2015;11(8):e1004337 10.1371/journal.pcbi.1004337 26291446 PMC4546376

[27] Bhat N, Wright JG, Broder KR, Murray EL, Greenberg ME, Glover MJ, et al Influenza-associated deaths among children in the United States, 2003–2004. N Engl J Med. 2005;353: 2559–2567. 10.1056/NEJMoa051721 16354892

[28] Miller E, Hoschler K, Hardelid P, Stanford E, Andrews N, Zambon M. Incidence of 2009 pandemic influenza A H1N1 infection in England: a cross-sectional serological study. The Lancet. 2010;375: 1100–1108. 10.1016/S0140-6736(09)62126-7 20096450

[29] Poletti P, Ajelli M, Merler S. The Effect of Risk Perception on the 2009 H1N1 Pandemic Influenza Dynamics. PLOS ONE. 2011;6: e16460 10.1371/journal.pone.0016460 21326878 PMC3034726

[30] Keeling MJ, Woolhouse ME, Shaw DJ, Matthews L, Chase-Topping M, Haydon DT, et al Dynamics of the 2001 UK foot and mouth epidemic: stochastic dispersal in a heterogeneous landscape. Science. 2001;294: 813–817. 10.1126/science.1065973 11679661

[31] Datta S, Bull JC, Budge GE, Keeling MJ. Modelling the spread of American foulbrood in honeybees. J R Soc Interface R Soc. 2013;10: 20130650. 10.1098/rsif.2013.0650 PMC3785836 24026473

[32] Gibson GJ. Investigating mechanisms of spatiotemporal epidemic spread using stochastic models. Phytopathology. 1997;87: 139–146. 10.1094/PHYTO.1997.87.2.139 18945133

[33] Neri FM, Cook AR, Gibson GJ, Gottwald TR, Gilligan CA. Bayesian analysis for inference of an emerging epidemic: citrus canker in urban landscapes. PLoS Comput Biol. 2014;10: e1003587 10.1371/journal.pcbi.1003587 24762851 PMC3998883

[34] Simini F, González MC, Maritan A, Barabási A-L. A universal model for mobility and migration patterns. Nature. 2012;484: 96–100. 10.1038/nature10856 22367540

[35] Schrödle B, Held L, Rue H. Assessing the impact of a movement network on the spatiotemporal spread of infectious diseases. Biometrics. 2012;68: 736–744. 10.1111/j.1541-0420.2011.01717.x 22171626

[36] Geilhufe M, Held L, Skrøvseth SO, Simonsen GS, Godtliebsen F. Power law approximations of movement network data for modeling infectious disease spread. Biom J Biom Z. 2014;56: 363–382. 10.1002/bimj.201200262 24843881

[37] Ferguson NM, Cummings DAT, Fraser C, Cajka JC, Cooley PC, Burke DS. Strategies for mitigating an influenza pandemic. Nature. 2006;442: 448–452. 10.1038/nature04795 16642006 PMC7095311

[38] Lindstrom MJ. Penalized estimation of free-knot splines. J Comput Graph Stat. 1999;8: 333–352.

[39] Diggle PJ, Kaimi I, Abellana R. Partial-likelihood analysis of spatio-temporal point-process data. Biometrics. 2010;66: 347–354. 10.1111/j.1541-0420.2009.01304.x 19673863

[40] Fonville JM, Wilks SH, James SL, Fox A, Ventresca M, Aban M, et al Antibody landscapes after influenza virus infection or vaccination. Science. 2014;346: 996–1000. 10.1126/science.1256427 25414313 PMC4246172

[41] Smith DJ, Lapedes AS, de Jong JC, Bestebroer TM, Rimmelzwaan GF, Osterhaus ADME, et al Mapping the antigenic and genetic evolution of influenza virus. Science. 2004;305: 371–376. 10.1126/science.1097211 15218094

[42] CDC. Influenza (flu) including seasonal, avian, swine, pandemic, and other. In: Centers for Disease Control and Prevention [Internet]. 29 4 2016 [cited 29 Apr 2016]. Available: http://www.cdc.gov/flu/index.htm

