# Supplementary material for: Human mobility and the spatial transmission of influenza in the United States
Source: PLoS Comput Biol. 2017 Feb 10;13(2):e1005382. doi: 10.1371/journal.pcbi.1005382 (PMC5349690; doi:10.1371/journal.pcbi.1005382)
Supplement: S1 Text — (PDF) [file pcbi.1005382.s001.pdf]

Human mobility and the spatial transmission of influenza in the United States

Supporting Text S1

Vivek Charu[1,2], Scott Zeger[2], Julia Gog[1,3], Ottar Bjørnstad[1,4], Stephen Kissler[3], Lone Simonsen[1,5],Bryan Grenfell[1,6], Cécile Viboud[1]

1. Fogarty International Center, National Institutes of Health, Bethesda, MD, USA
2. Department of Biostatistics, Johns Hopkins Bloomberg School of Public Health, Baltimore, MD, USA
3. Department of Applied Mathematics and Theoretical Physics, University of Cambridge, Cambridge, UK
4. Department of Entomology, Pennsylvania State University, State College, Pennsylvania, United States of America,
5. Department of Public Health, University of Copenhagen, Copenhagen, Denmark
6. Department of Ecology and Evolutionary Biology, Princeton University, Princeton, NJ, USA

Here we provide supplementary data and sensitivity analyses to evaluate the robustness of our findings.

A. Analyses of influenza dominant subtypes, epidemic onsets, long-range transmission events, epidemic origins and the role of spatial aggregation

1. *Influenza subtype dominance, 2002/03-2009/10*. Subtype prevalence varied across seasons, with 5 of the 8 seasons dominated by a single subtype, representing more than 75% of all influenza positive isolates. See Table A1.

2. *Agreement of epidemic onset times determined by piecewise spline method and traditional Serfling regression*. In this paper we have proposed a straightforward method for obtaining estimates of epidemic onset in each geographic location. The proposed piecewise spline method has advantages over traditional time series harmonic regression models in that it (i) can be applied to short time series (one epidemic cycle, instead of typically ≥3 cycles/years for time series regression) and (ii) provides a clear approach to estimating the uncertainty in onset times via the curvature of the profile likelihood (see Figure A1 as an illustration). In Figure A12, we compare onset estimates derived using the piecewise approach with the more traditional Serfling harmonic regression models applied to non-epidemic periods (see (1–4) for a more complete description). Overall, there was excellent agreement between the two methods. For onset times that were highly mismatched between the two methods visual inspection of time series indicated that the harmonic regression approach occasionally identified spurious short-lived excursions above the seasonal baseline, while the piecewise spline method of the main analysis did not suffer from this drawback.

3. *Long-range transmission events*. We proposed an empirical method to identify long-range influenza transmission events. This method relies on identifying outliers in the distribution of distances between newly infected cities and the set of infectious cities at the previous time step (see methods in main text for further details). Figure A3 plots a histogram of this distribution, and Table A2 provides further details on the number of long-range events identified in each season.

4. *Estimating the geographic origin of each epidemic*. To identify the geographic origin of the epidemic in each season, we adapted the "effective distance" method of Brockmann *et al*. (5) using geographic distance as the "effective distance" between pairs of locations. Figure A4 displays the relationship between onset times in each location and distance from the estimated origin of the epidemic. Based on the method in Brockman et al. (5), the presumed origin is the location that maximizes the correlation between onsets and effective distance, over all possible origin locations.

5. *Role of spatial aggregation and spatial synchrony*. To gauge the effect of the choice of methodology used to estimate spatial patterns in epidemic data, and to facilitate comparison with previous work, we applied the same methodology proposed in Viboud et al. (1) to our city-level medical claims data. Briefly, we computed pairwise correlations in weekly ILI incidence over the 8-year study period as a measure of synchrony in the amplitude of city-level epidemics. Further, we computed pairwise correlation in wavelet-derived ILI phase estimates as a measure of correlation in epidemic timing. A non-parametric spatial covariance function (6) was applied to smooth and quantify spatial patterns. We then used Mantel tests to assess the role of geographic distance, population size, work commutes, and air traffic on spatial synchrony. Finally, to assess the effect of the level of spatial aggregation on estimated spatial patterns, we repeated these analyses with disease data aggregated at the state level.

   We found that the effect of geographic distance was more pronounced at the city level, relative to state, while the effect of population size weakened at the city level (Table A6, Figures A10, A11). Importantly, synchrony analysis of state-level ILI data recovered the strong hierarchical patterns of spread identified in Viboud et al. (1) based on state-specific mortality time series, with both analyses demonstrating marked effects of distance and population size on influenza synchrony (Table A6). In Mantel analyses, work commutes were the strongest predictors of synchrony at the state level in univariate and adjusted analyses. Interestingly, the effect of work commutes substantially decreased at the city level, so that geographic distance became the main driver of synchrony in city-level data. Hence city-level synchrony analyses align with our main analysis and support a primarily localized mode of influenza transmission driven by geographic distance.

   Overall, these sensitivity analyses capturing synchrony in full epidemic time series rather than limited to epidemic onset times, confirm the robustness of the spatial effects evidenced in the main text and validate earlier work (1). The analyses also suggest that spatial aggregation may introduce particular biases, such as attenuation of distance effects and exacerbation of demographic effects. Further research is warranted to evaluate how data aggregation may affect our understanding of the spatial transmission of infectious diseases.

B. Sensitivity analyses on spatial transmission model.

1. *Model performances under complete spatial randomness*. We sought to explore the distributions of parameter estimates in the setting of complete spatial randomness. To do so, we generated 500 artificial datasets in which we permuted the influenza onset times between locations in a single epidemic, using the strongly spatial 2003-04 season as an illustration. For each simulated dataset, we re-estimated the model. Summary statistics for each parameter are

shown in Table A7 and indicate that the model is well behaved, in that it does not detect spatial effects when there are none.

2. *Model performance under realistic uncertainties in epidemic onsets*. The model presented in the main text relies on the assumption that influenza epidemic onset times are known. We sought to explore the robustness of parameter estimates from the model in the presence of realistic uncertainties in the onset times, where uncertainty is based on the profile likelihood resulting from the piecewise spline onset estimation method. To do so, we generated 500 datasets in which epidemic onset times were drawn from a normal distribution centered at the estimated onset time $\widehat{t_j}$, with variance $\widehat{\sigma_j}^2$. In Figure A13 we display results from this procedure applied to data from the 2003/2004 epidemic and indicates that the estimates presented in the main text are robust to uncertainty in onset dates.

3. *Parameter estimates from a more complex model*. For each season, we fit a fuller model than in the main analysis by incorporating information on absolute humidity and the population size of the infectious city. Parameter estimates are provided in Table A3. Parameter estimates associated with absolute humidity overlap zero in all seasons while those associated with donor population size overlap zero in 7 of the 8 seasons. Further the sign of the estimates is not consistent across seasons, lending further support to the absence of an association. Reassuringly, the addition of these variables has little impact on the other estimates.

4. *Role of normalization factor and population size on risk of transmission*. Figure A7 maps how baseline susceptibility, $N_j^{\mu}(\sum_{k:k\neq j} d_{jk}^{\gamma})^{-\varepsilon}$, varies throughout the network of cities and reveals that the normalization factor, $(\sum_{k:k\neq j} d_{jk}^{\gamma})^{-\varepsilon}$, inflates influenza risk in remote cities (those with fewer neighbors).

5. *Comparison of full and partial likelihood approaches, and time-dependence in the baseline risk of infection*. To gauge the importance of temporal variation in the hazard of infection throughout the course of an epidemic, we used a fully-parametric model as in previous work and considered two scenarios for the baseline risk of transmission: (A) a constant baseline hazard and (B) a time-varying cubic baseline hazard. Table A5 presents AIC values from the two models, indicating that in all seasons except 2005/2006 epidemic, time varying baseline hazards outperform simple constant baseline hazards. As an illustration, Figure A8 displays the shapes of the baseline hazards over time for each season (assumed to be cubic in time). Using the 2003-04 epidemic as a case study, a plot of simulated trajectory of the epidemic against the observed data demonstrates why time-varying baseline hazards are important for capturing the temporal aspects of epidemic dynamics (Figure A9).

In addition to testing the robustness of our analysis to the shape of the baseline hazard, we also assessed the importance of the estimation approach. To

do so, we compared results from the partial likelihood approach used in the main text (inspired by the FMD transmission model by Diggle et al. (7,8)) and the full likelihood approaches. This sensitivity analysis displays general agreement between the two approaches in terms of parameter estimates (Table A4). In particular, parameter estimates for the distance-based model in the 2009 pandemic align extremely well with those reported by Gog et al. (3) ($\hat{\gamma} = -2.6, \hat{\varepsilon} = 0.9, \hat{\mu} = 0.3$; this study: $\hat{\gamma} = -2.6, \hat{\varepsilon} = 1.2, \hat{\mu} = 0.2$), despite use of a different method for determining influenza onset times, a different model formulation, and a different objective function for maximization (partial likelihood v. full likelihood). The agreement between the two studies confirms the robustness of the findings on the 2009 pandemic while also validating the partial-likelihood approach, suggesting that loss of information about spatial parameters by considering only the relative ordering of onset times (as opposed to the onset times themselves) is minimal.

6. *Model with external seeding constrained to zero*. To compute a comparative summary measure of the spatial diffusiveness of each epidemic, we refit the semi-parametric models while constraining the external seeding term ρ to 0. This nested model, though missing an important mechanistic component of the spatial process, allows for the distance power-law exponent γ to act as an index of spatial diffusiveness; more negative values of γ indicate more locally diffusive epidemics. Point estimates of γ under this model indicate that the 2003/2004 and 2005/2006 epidemics along with the 2009 pandemic were characterized by more locally diffusive spread ($\hat{\gamma} = 2.10$ to 2.36) compared to other seasons ($\hat{\gamma} = 1.76$ to 1.89) (Table A8).

7. *Understanding differences in the age structures of populations across US counties*. Estimates for the parameter μ suggest a moderate increase in a city's risk of influenza infection with increasing population size; differences in the age structure between small and large populations could confound this relationship. To explore this idea, we obtained data on the population in each of 3142 counties in the US stratified by age, from the 2010 census (https://www.census.gov/popest/data/counties/asrh/2015/CC-EST2015-ALLDATA.html). We studied the proportion of the population in four age strata (under 20yrs, 20-50yrs, 50-65yrs and over 65yrs) and find that more populous counties have larger proportions of their populations made up by 20-50 year olds, presumably a more mobile segment of the population (Figure A14).

C. Supplementary discussion on the role of the air traffic and work commute networks, relative to geographic distance, on the spatial transmission of influenza

1. *Degree distribution of airline traffic and work commute matrices*. Figure A5 plots the degree distribution of the air traffic and work commute networks. The

degree distribution quantifies the number of connections emanating out of each node in the network, here 306 counties in our dataset. We find that the air traffic network is less sparse than the work commute network, while any two cities in the network are connected by geographic distance (and thus the degree distribution is constant at 306). This indicates that a substantially fewer number of possible epidemic pathways are allowed by a model driven by work commutes, likely contributing to the poorer fit of models driven by work commutes compared to geographic distance.

2. *Understanding the relationship between work commutes and geographic distance*. In order to allow conceptual comparisons between models utilizing different human mobility indices, we put the work commutes and air traffic networks on the same "scale" as that of geographic distance by estimating the relationships between the networks. Figure A6 depicts these relationships. These analyses indicate that work commutes scale with geographic distance according to a power law of ~3.3 for the counties within 119km of each other, as sampled in our dataset. Because influenza transmission is estimated to scale according to a power-law of 2.2 with distance (main text), this indicates that influenza transmission is expected to scale with work commutes according to a power-law of 2.2/3.3 = 0.67. It is very reassuring to note that the expected value of 0.67 is almost identical to the median value of the scaling parameter for work commutes estimated for the influenza transmission models using commutes as connectivity between locations (main text). Thus we hypothesize that the power-law parameter associated with work commutes in the transmission model acts to tune the work-commute connectivity matrix to make it "less" localized, and aid its ability to capture the appropriate geographic scale of influenza transmission, which is somewhat less localized than pure US work commutes. This reasoning also supports the internal consistency of flu transmission model estimates.

Table A1. Influenza virus distribution each season, 2002-2010. The table lists the percent of influenza specimens by type and subtype each season (so that the proportion of A and B sums to 100% each season). Data are based on the World Health Organization and the National Respiratory and Enteric Virus Surveillance System Collaborating Laboratories; abstracted from CDC summary reports each season (9). Dominant (sub)type(s) are highlighted in bold (the dominant subtype is commonly defined as the subtypes (or top two subtypes) representing more than 75% of all influenza specimens isolated in a given season).

Season	Influenza A	Influenza B	Influenza A subtypes:		
			A/H3N2	A/H1	H1N1pdm
2002/2003	**56.40%**	**43.60%**	29.70%	**70.30%**	0.00%
2003/2004	**99.00%**	1.00%	**99.90%**	0.10%	0.00%
2004/2005	**75.40%**	24.60%	**99.70%**	0.30%	0.00%
2005/2006	**79.70%**	20.30%	**91.90%**	8.10%	0.00%
2006/2007	**79.20%**	20.80%	**37.70%**	**62.30%**	0.00%
2007/2008	**71.00%**	**29.00%**	**74.00%**	26.00%	0.00%
2008/2009	**66.00%**	**34.00%**	13.00%	**87.00%**	0.00%
2009/2010	**99.00%**	1.00%	4.00%	2.00%	**94.00%**

Table A2. Summary statistics of the (d-D) distribution for each season, which helps quantify the spatial structure of each epidemic. The 2003/2004 epidemic and 2009 pandemic had more local modes of transmission than other seasons, as illustrated by a low mean d-D. The last 2 columns identify the number of long-range transmission events each season for two distance thresholds based on the overall d-D statistics (see also Figure S3).

(d-D) Seasonal Summary Statistics								
**Season**	**Min**	**1st quartile**	**Median**	**Mean**	**3rd quartile**	**Max**	**N>99th %**	**N>97.5th %**
**2002/2003**	0	0	0	64.4	38.74	1639	2	6
**2003/2004**	0	0	0	**52.76**	43.65	1775	1	6
**2004/2005**	0	0	0	72.63	47.99	3863	5	8
**2005/2006**	0	0	5.467	69.84	69	2045	2	4
**2006/2007**	0	0	0	71.54	59.85	1167	3	8
**2007/2008**	0	0	2.416	66.77	47.42	2139	3	7
**2008/2009**	0	0	0	80.95	60.33	1223	2	5
**2009/2010**	0	0	1.822	**55.27**	47.83	2755	2	6

Table A3. Parameter estimates from a spatial model incorporating terms for absolute humidity in the previous two weeks before onset, $\hat{\nu}$, and donor population size, $\hat{\phi}$, **using the partial likelihood approach**. In 2006/2007 the full model was not estimable, so we excluded the absolute humidity parameter ($\hat{\nu}$) and refit the model. In all seasons, the effect of absolute humidity and donor population size is not significant (except for the 2007/2008 season, for donor population size). Further the sign of the estimates is not consistent across seasons, lending additional support to the absence of an association. Reassuringly, the addition of these covariates had little impact on the other parameter estimates. Values in parentheses indicate the standard deviation of parameter estimates.

Parameter	2002/2003	2003/2004	2004/2005	2005/2006	2006/2007	2007/2008	2009/2010
distance (km) (γ)	2.03 (0.2)	2.26(0.16)	2.38(0.2)	2.25(0.13)	2.52(0.31)	1.95(0.22)	2.67(0.15)
recipient pop size (μ)	0.08 (0.07)	0.23(0.06)	0.35(0.08)	0.28(0.07)	0.34(0.07)	0.26(0.07)	0.18(0.07)
normalization (ϵ)	0.57 (0.09)	1.07(0.06)	0.98 (0.06)	0.80(0.08)	0.59(0.04)	0.82(0.08)	1.15(0.05)
external seeding (log (ρ))	-8.86 (0.95)	-1.86(0.82)	1.36(0.43)	-5.86(0.96)	-1.16(1.92)	-5.25(0.68)	-0.23(0.47)
external seeding (ρ)	0.00014 (0.00013)	0.16 (0.13)	3.9 (1.67)	0.0029 (0.0028)	0.31 (0.5952)	0.0052 (0.0035)	0.79 (0.37)
absolute humidity ($\hat{\nu}$)	-0.19(0.28)	-0.03(0.22)	0.08(0.23)	0.26(0.27)	-	-0.23(0.25)	-0.29(0.18)
donor pop size ($\hat{\phi}$)	0.08(0.14)	-0.09(0.15)	-0.16(0.15)	0.42(0.21)	-0.26(0.16)	0.40(0.17)	0.006(0.24)

Table A4. Comparison of maximum partial likelihood estimates with maximum likelihood estimates. **Fully-parametric models are fit using the discrete-time formulation in which epidemic onsets occur in half-week intervals as in (3). Here we report on the cubic baseline hazard models, which, in most seasons, are better fit to the data than models assuming constant baseline hazards (see Table S5).

Seasons:	**2002/2003**	**2003/2004**	**2004/2005**	**2005/2006**	**2006/2007**	**2007/2008**	**2009/2010**
Partial Likelihood							
distance (γ)	2.08	2.22	2.30	2.19	2.65	2.18	2.64
recipient pop size (μ)	0.11	0.21	0.35	0.31	0.33	0.28	0.15
normalization (ϵ)	0.59	1.06	0.96	0.86	0.62	0.91	1.16
external seeding (log (ρ))	-6.56	-3.47	-0.013	-19.4	-2.48	-1.01	-1.29
external seeding (ρ)	0.0014	0.031	0.99	3.8E-9	0.084	0.36	0.28
Full Likelihood, cubic baseline hazard							
distance (γ)	1.95	1.88	2.00	2.34	2.09	1.74	2.25
recipient pop size (μ)	0.20	0.01	0.18	0.27	0.34	0.21	-0.01
normalization (ϵ)	0.59	0.73	0.82	0.7	0.36	0.5	1.05
external seeding (log (ρ))	-5.27	-6.29	-4.86	-7.04	-5.32	-6.08	-5.66

Table A5. Comparison of AIC values from spatial models assuming a cubic baseline hazard compared to those assuming a constant baseline hazard. Note that in the main text, we use a partial likelihood approach, which does not presume any shape for the baseline hazard (it is treated as a nuisance parameter). To produce the table below, we use a full likelihood approach as in (3), assuming constant and cubic functions of time for the baseline hazard function. These results indicate that, in most seasons, the hazard of infection changes throughout the epidemic (see also Figures S8 and S9). Bold values indicate stronger statistical support (lower AIC).

**Season**	**AIC**	
	**Spatial model with cubic baseline hazard**	**Spatial model with constant baseline hazard**
2002/2003	**1278.64**	1295.03
2003/2004	**1374.12**	1431.38
2004/2005	**1496.62**	1556.22
2005/2006	1371.26	**1368.0**
2006/2007	**1345.53**	1405.05
2007/2008	**1296.69**	1306.91
2008/2009	**-**	-
2009/2010	**1599.74**	1672.93

Table A6: Association between ILI activity, distance, and mobility indicators at two levels of spatial aggregations (city and state), using the Mantel statistics (1, 6).

Epidemic indicator	Geographic and mobility indicators	*State level*		*City level*	
ILI rates	Distance	-0.35 (P<0.001)	NS	**-0.35 (P<0.001)**	**-0.36; -0.33 (P<0.001)**
	Workflows	**0.51 (P<0.001)**	**0.19-0.49 (P<0.003)**	0.22 (P<0.001)	NS
	Air traffic	0.20 (P=0.024)	NS	0.17 (P<0.001)	NS
ILI phases	Distance	-0.14 (P=0.05)	NS	**-0.17 (P<0.001)**	**-0.15; -0.16 (P<0.001)**
	Workflows	**0.48 (P<0.001)**	**0.22-0.45 (P<0.001)**	0.10 (P=0.09)	NS
	Air traffic	0.25 (P=0.012)	NS	0.04 (P<0.001)	NS

Table A7. Approximate range of parameter estimates under the hypothesis of complete spatial randomness for a single epidemic. Estimates were generated for each of 500 permutations of influenza onset times in the 2003/2004 epidemic. Note that we cannot compare univariate parameter estimates here with those presented in the main text as the parameters are highly correlated (especially γ, ϵ and ρ).

Parameter	Median (1st-3rd quartiles)
γ	1.06 (0.42 to 1.9)
μ	-1.17 (-3.23 to -0.18)
ϵ	0.52 (-0.53 to 1.00)
$\log(\rho)$	-0.3501 (-2.34 to 0.27)
ρ	0.705 (0.096 to 0.763)

Table A8. To compute a comparative summary measure of the spatial diffusiveness of each epidemic, we refit the semi-parametric models while constraining the external seeding term ρ to 0: $\lambda\left(t_j \middle| \mathcal{H}_{t_j}, \mu, \gamma, \varepsilon\right) = \lambda_0(t_j)[N_j^{\mu} \sum_{i \in I_{t_j}} \frac{d_{ij}^{-\gamma}}{(\sum_{k:k \neq j} d_{jk}^{-\gamma})^{\varepsilon}}]$. This nested model, though missing an important mechanistic component of the spatial process, allows for the distance power-law exponent γ to act as an index of spatial diffusiveness; more negative values of γ indicate more locally diffusive epidemics. Parameter estimates and standard errors are provided in the table below. The most negative values of γ are highlighted in bold.

Season	γ	SD	μ	SD	ϵ	SD
**2002/2003**	1.87	0.13	0.089	0.067	0.55	0.093
**2003/2004**	**2.10**	0.12	0.217	0.062	1.04	0.063
**2004/2005**	1.90	0.14	0.292	0.073	0.98	0.080
**2005/2006**	**2.19**	0.12	0.287	0.073	0.79	0.082
**2006/2007**	1.84	0.16	0.267	0.070	0.60	0.14
**2007/2008**	1.76	0.13	0.292	0.065	0.87	0.083
**2008/2009**	1.76	0.20	0.409	0.10	0.89	0.092
**2009/2010**	**2.37**	0.12	0.157	0.063	1.10	0.055

Figure A1. Illustration of algorithm used to estimate epidemic onset. The panel on the left displays the ILI time series for Aberdeen, SD during the 2003/2004 epidemic, which is one of the smaller locales in our sample of 310 cities. The algorithm fits a piecewise-linear function to the pre-epidemic period, and to the epidemic ascending phase (up to the peak), and determines the most likely breakpoint that corresponds to a switch to epidemic mode (blue line = epidemic onset estimate). The highlighted portion in red indicates the section of the time series used to determine the epidemic onset. The panel on the right displays the profile likelihood for the epidemic onset time, as a function of the other parameters. The maximum of the profile likelihood is identified as the blue dashed line (estimate of epidemic onset =week 26.5).

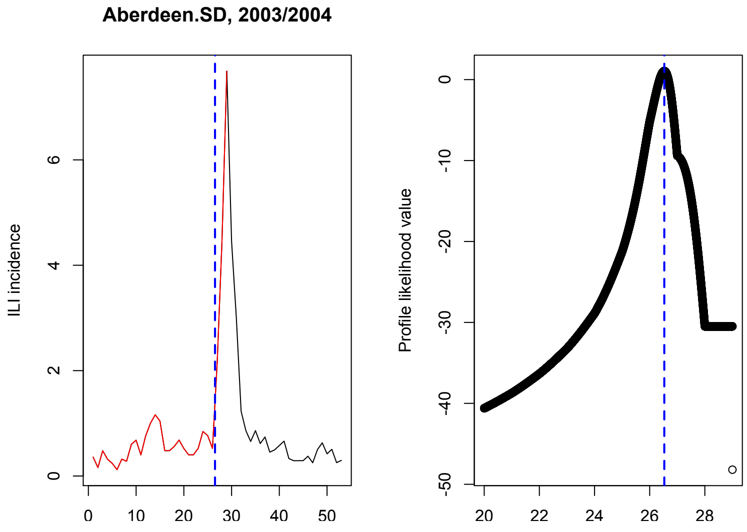

Figure A2: Influenza onset times across eight seasons, 2002/2003-2009/2010 at four different time points in each season. Colored circles represent infected locations at the current time point, while grey circles represent locations infected at a previous time point.

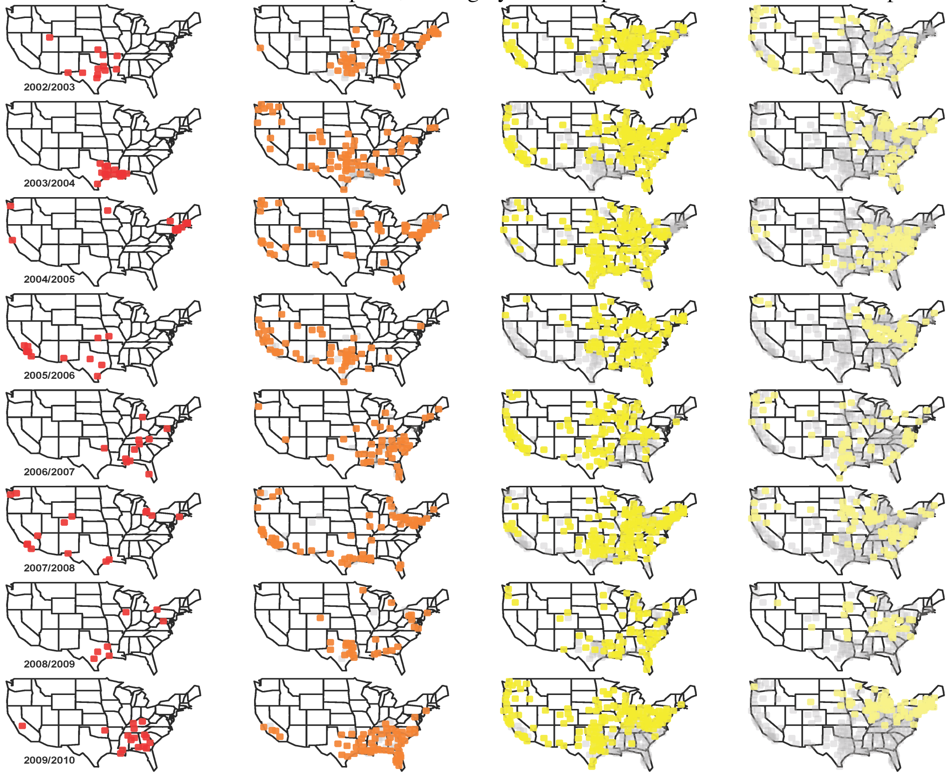

Figure A3. **Long-range transmission events.** Below is the distribution of d-D across eight seasons considered in this study. The dashed blue line is the 97.5th percentile (corresponding to 525.5 km) and the dashed red line is the 99th percentile (corresponding to 940.9 km). Locations falling in the 1st percentile of this distribution were considered to be infected via long-range transmission.

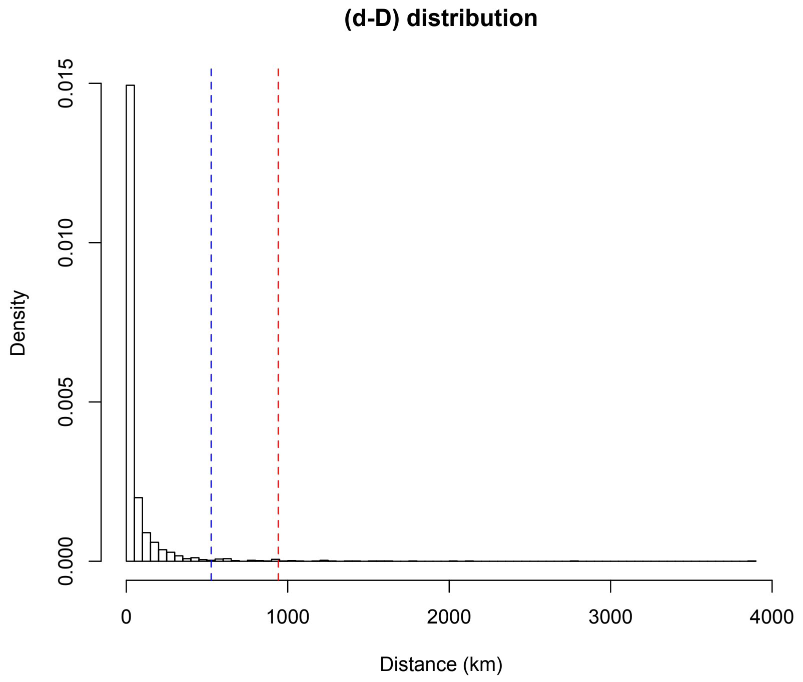

Figure A4. **Relationship between influenza onset times (y-axis) and distance to most likely source locations (x-axis) in each season.** An algorithm similar to Brockman et al (6) was used to identify the most likely origin of the epidemic each season, as the location that should maximize the correlation of onset times with distance to said location. In each panel, the identified source location and correlation coefficient are provided at the bottom right. The spatial relationship is particularly strong in 2002/2003, 2003/2004, 2005/2006, and 2009/2010, in line with the analyses presented in Figure 2 of the main text.

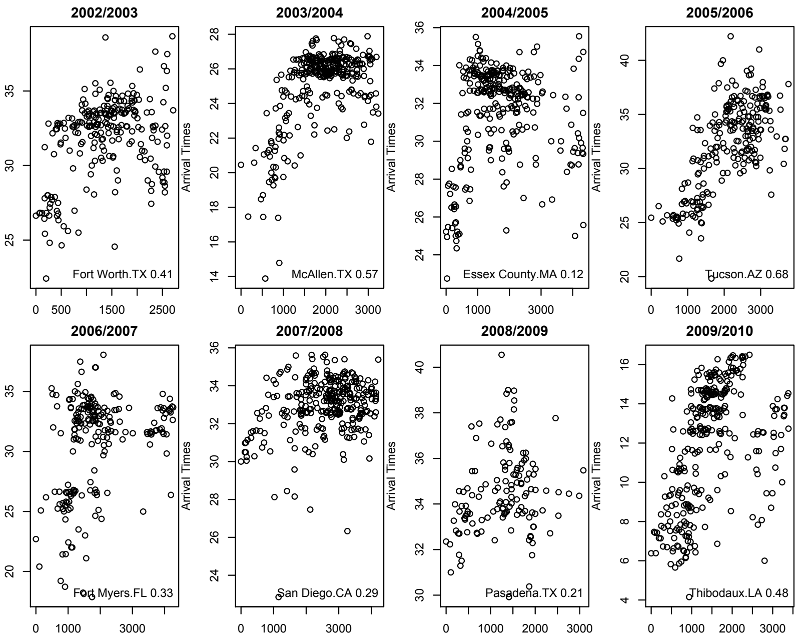

Figure A5: The degree distributions of the work commute and air traffic matrices. These distributions reflect the number of connections each location has based on work commute and air traffic statistics. The dashed black lines are the median degrees (54 for the work commute matrix and 136 for the air traffic matrix), and the dashed blue lines are the mean (64.2 v. 112.9). This indicates that on average, each location has more connections via air travel* than via work commutes (despite this, the commute-based models fit the data significantly better than the air travel models). Note that the network of cities is fully connected when using the geographic distance metrics.
*This is in part because we consider all airports within 100km of each locale to build the air traffic matrix

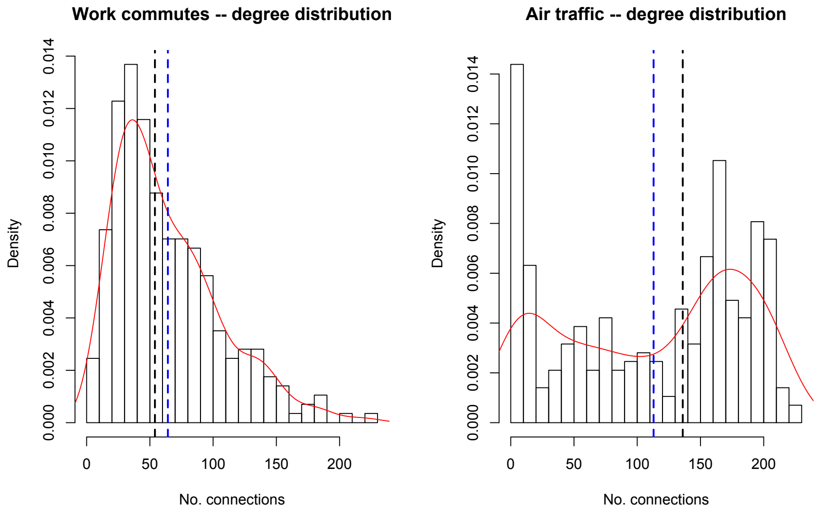

Figure A6: Estimating the relationships between pairwise work commutes, air traffic and geographic distance. In order to allow conceptual comparisons between models utilizing different human mobility indices it is important to understand how different metrics of human mobility scale with geographic distance. Here we fit power-law type models using symmetrized workflow and air traffic data. Letting W_{ij} and A_{ij} be the symmetrized work commutes and air traffic fluxes between locations *i* and *j*, the relationships: $W_{ij} = \beta_w d_{ij}^{\gamma_w}$ and $A_{ij} = \beta_a d_{ij}^{\gamma_a}$, imply the loglinear forms: $logW_{ij} = log\beta_w + \gamma_w logd_{ij}$ and $logA_{ij} = log\beta_a + \gamma_a logd_{ij}$. For workflows, we estimate parameters separately for d_{ij} <119km and ≥119km as in Viboud et al (1). Parameter estimates were obtained using ordinary least squares (with robust standard errors) and are provided in the figure below. For comparison, Viboud et al. (1) report that asymmetric county-to-county workflows scale with geographic distance according to the following piecewise function: $W_{ij} \propto \begin{cases} d_{ij}^{-3.05} \; for \; d_{ij} < 119km \\ d_{ij}^{-0.29} \; for \; d_{ij} \geq 119km \end{cases}$. Data below are from 290 counties available for analysis in the 2003/2004 epidemic.

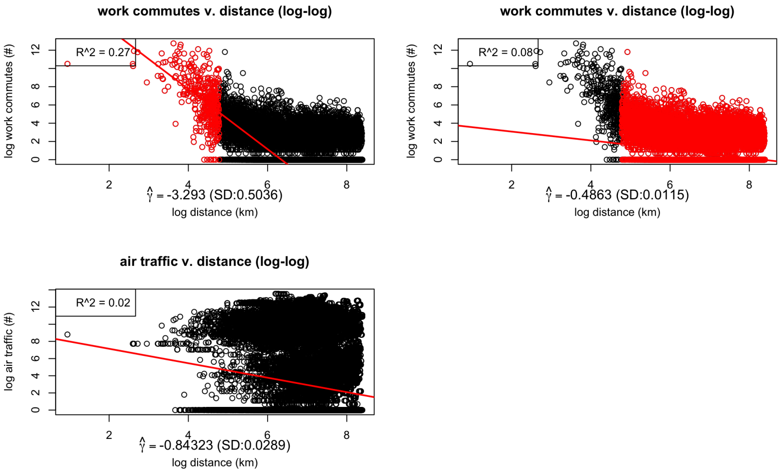

Figure A7. Understanding the distance normalization factor. The map below plots $(\sum_{k:k\neq j} d_{jk}^{-\gamma})^{-\varepsilon}$ (evaluated at median values, $\gamma = 2.2, \varepsilon = 0.9$), revealing how the estimated normalization factor varies throughout the network (the size of each circle in the map below is proportional to the estimated values of $(\sum_{k:k\neq j} d_{jk}^{-\gamma})^{-\varepsilon}$. In the density-dependent transmission model, $\varepsilon = 0$, implies $(\sum_{k:k\neq j} d_{jk}^{-\gamma})^{-\varepsilon} = 1$, and the normalization would be constant across cities in the network. The estimated normalization factor appears to inflate to inflate the estimated risk of influenza transmission in remote cities, especially in the Midwestern (a region under-sampled in our dataset), Southwestern and Northwestern US.

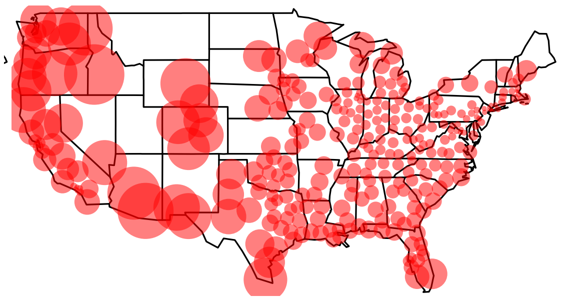

Figure A8: Shape of the estimated baseline hazards, when assumed to be cubic functions over time for six seasons. Depicted below are best-fit time-varying baseline hazards (assumed to be cubic in time) at each time step of the epidemic (x-axis, t in weeks, where t=0 represents the start of the epidemic in the location with earliest onset, y-axis is proportional to risk of transmission at time t). We exclude the 2005/2006 season where the constant baseline hazard model outperformed the cubic hazard (see Table S5), and the mild 2008/2009 season due to few observations.

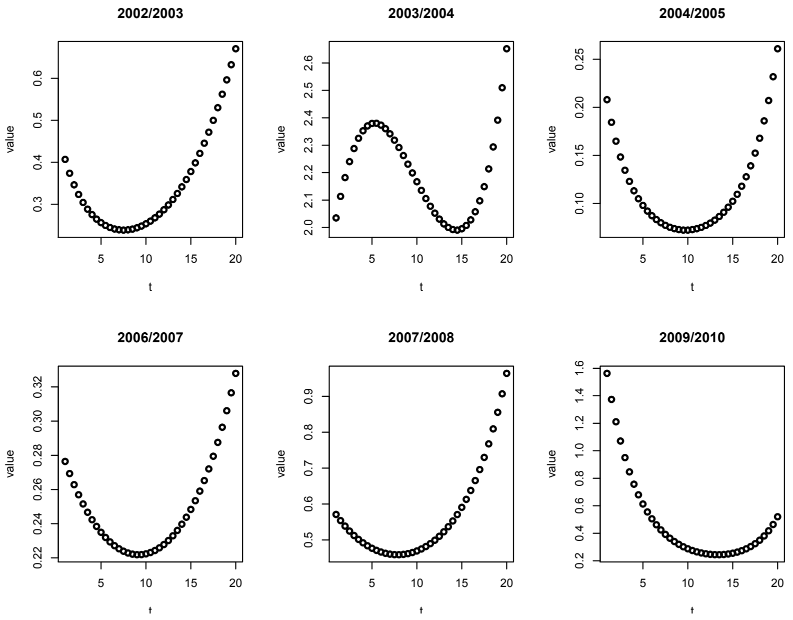

Figure A9. Comparison of observed v. simulated epidemics for spatial models assuming constant versus time-varying baseline hazards. These models are fit using maximum likelihood methods, as in (3). The red line indicates the observed epidemic trajectory (2003/2004 season), and each black lines is a model-based simulation of the epidemic process, using the maximum likelihood parameter estimates in Table S5. At time point ~16 (x-axis), it is clear that simulated epidemic trajectories from the time-varying baseline hazard model are able to better approximate the observed trajectory (in red) than those simulated using a constant baseline hazard, in line with the AIC values presented in Table S6. Use of a partial likelihood approach, as in the main text, alleviates the need to specify a form for the baseline hazard function.

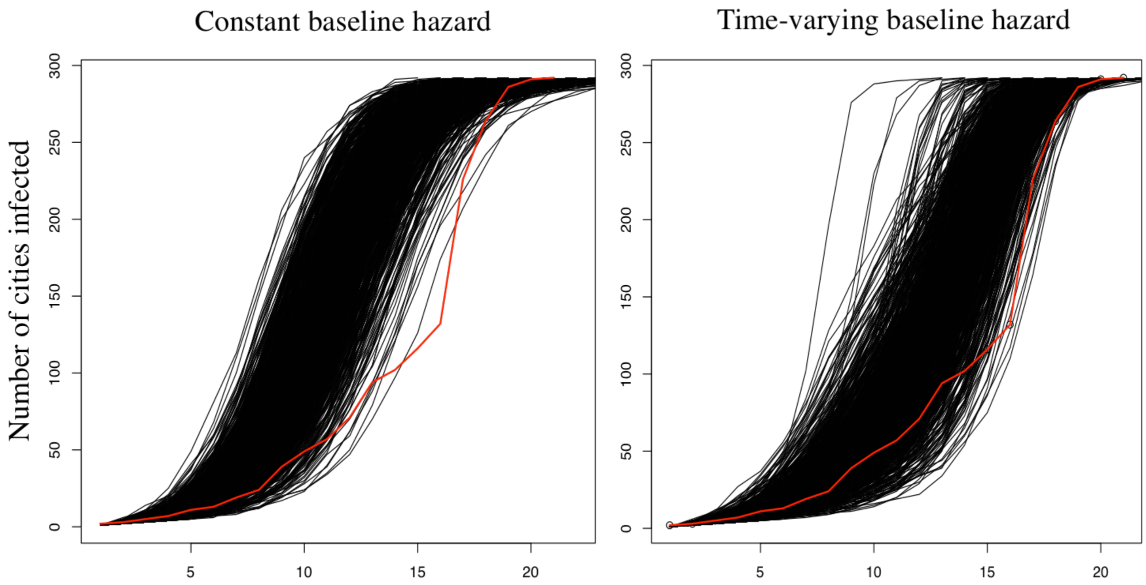

Figure A10. Synchrony and distance signature in ILI time series at two different spatial scales. Following the methodology proposed in Viboud et al. (1), the left panels display the pairwise correlation between weekly ILI rates (measuring amplitude and timing) and geographic distance between each pair of locations. The right panels display the pairwise correlation between weekly ILI phases (a measure of timing) and geographic distance between each pair locations. Top panels present correlations at the city level while bottom panels represent correlations at the state level. Curves are based on the NCF non-parametric smoothing package (6), including a spline fit (red line) and 95% CI (black lines). The horizontal dotted line represents the estimate for global synchrony (average correlation across all pairs of locations).

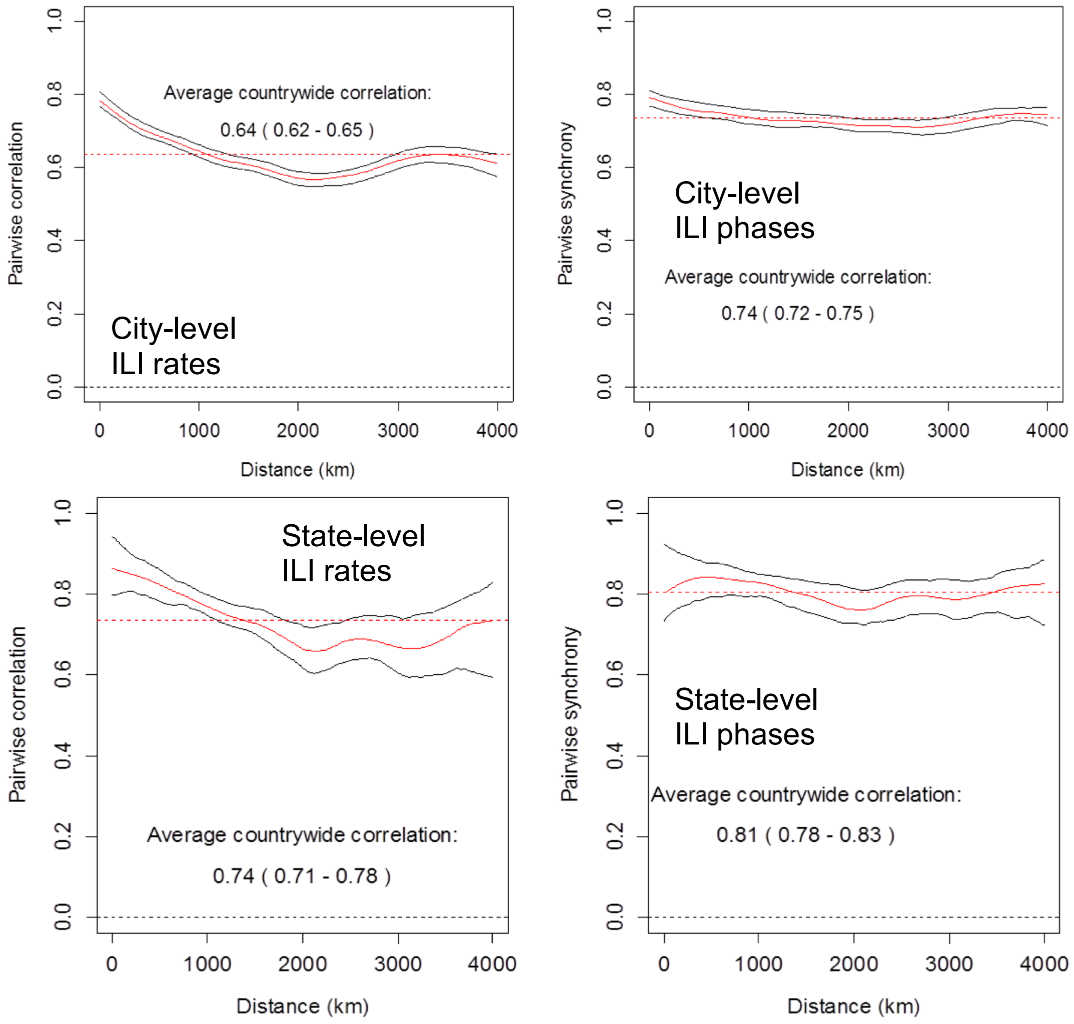

Figure A11: Comparison of the effect of population size on epidemic synchrony at two different levels of spatial aggregation (city-level, top panels; and state-level, bottom panels). Left panels: pairwise correlation in weekly ILI rates between locations, as a function of population size (organized by quartiles, ranging from very low populated locales on the left to very populous locales on the right, as in (1)). Right panels: same but with weekly phases. This indicates a hierarchical pattern of influenza spread in state-level data, consistent with previous work (1), which becomes attenuated in city-level data.

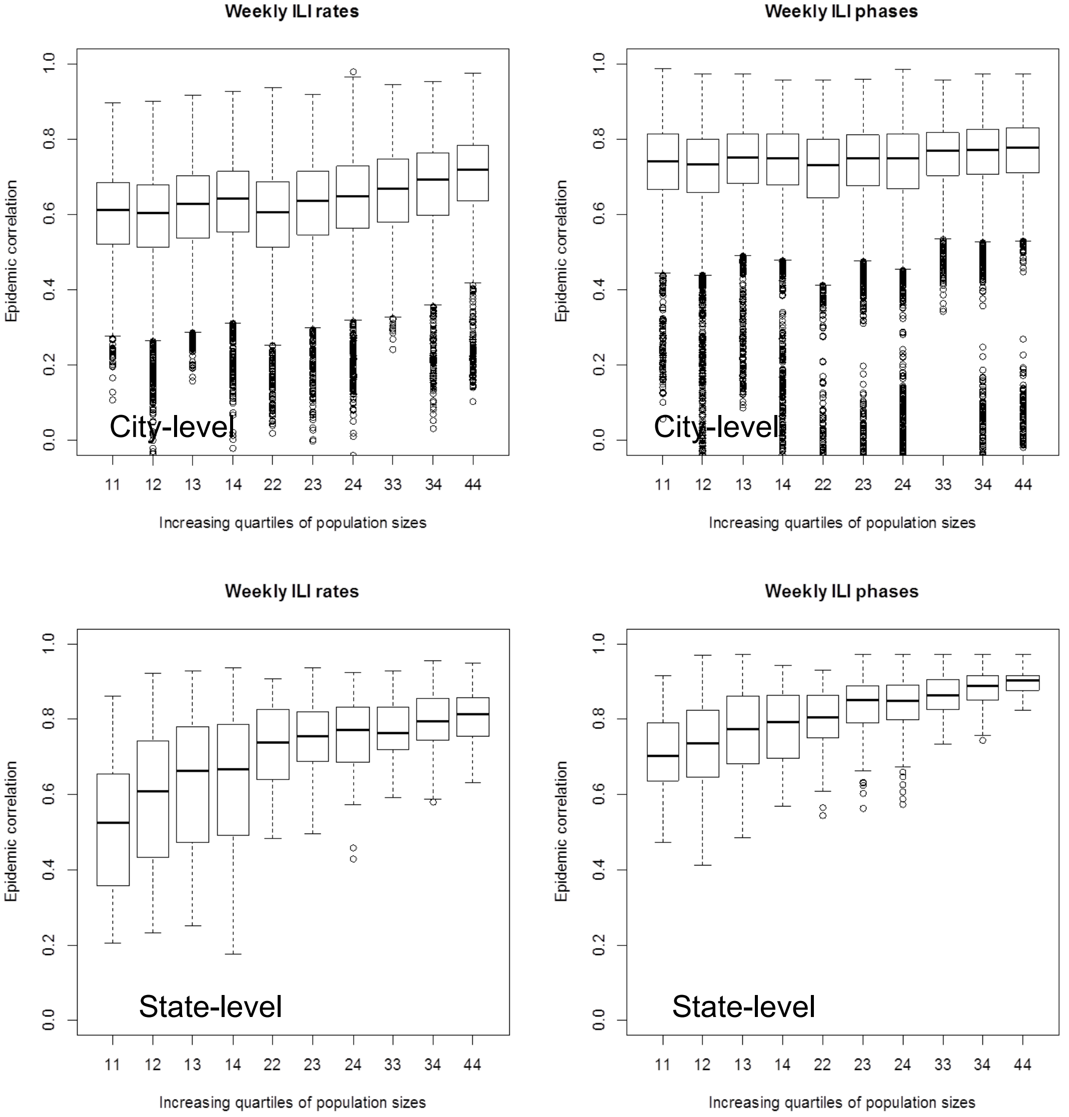

Figure A12: Agreement between estimates of epidemic onset times by two methods: a piecewise spline method (as in main text, see also Fig S3 for an illustration, x-axis) and a seasonal regression model based approach ("Serfling method", as in (1-4), y-axis). In general, there is excellent agreement between the two methods.

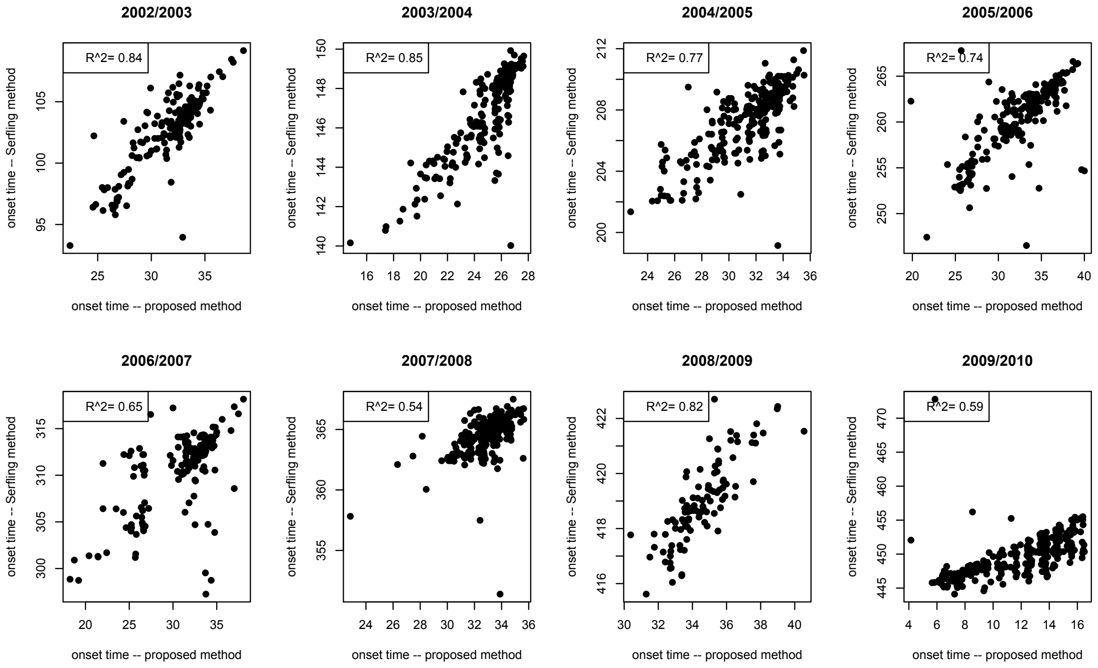

Figure A13. Parameter estimates for the spatial model for the 2003/2004 epidemic under the setting of moderate uncertainties in the epidemic onset times. The red dashed line represents the median across 500 datasets in which the epidemic onset times are drawn from a normal distribution centered at the estimated onset time $\widehat{t_J}$, with variance $\widehat{\sigma_J}^2$. The vertical blue are the parameter estimates presented in the main text (distance (γ), recipient pop size (μ), normalization (∈), external seeding (ρ, log scale))

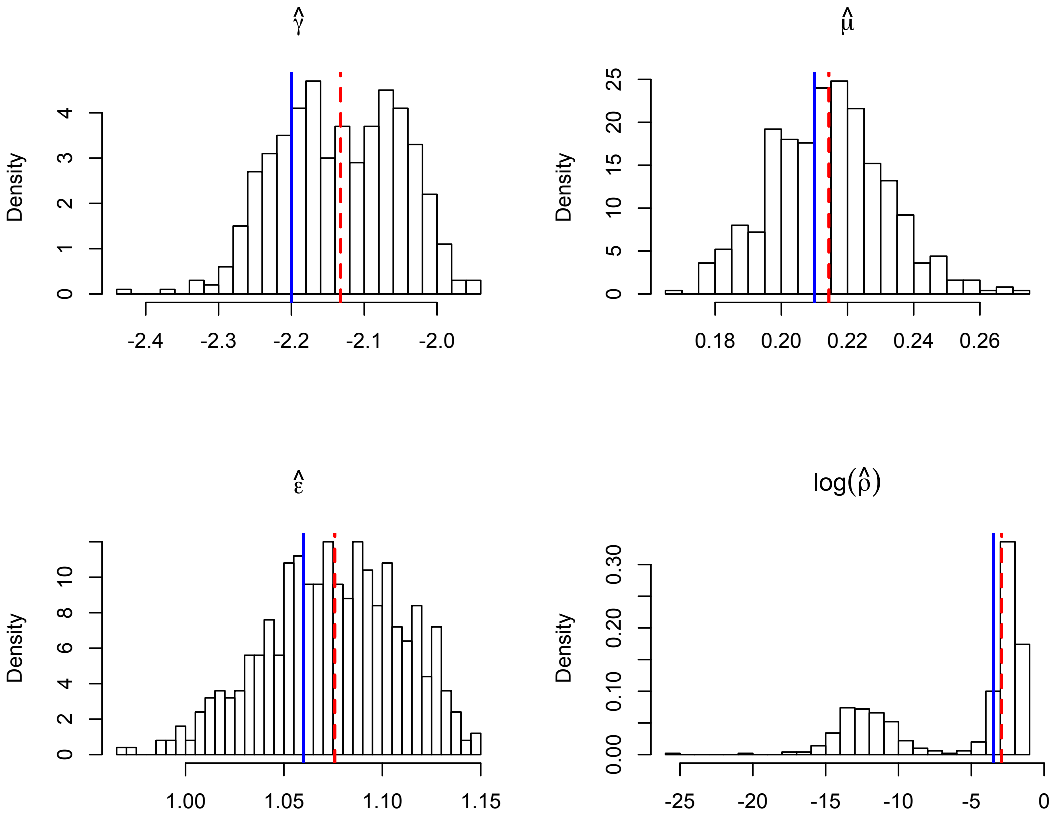

Figure A14. Differences in the age structures between small and large populations at the county level in the US. In each of the panels below, the x-axis provides the total population for each of 3142 counties in the US (log scale). The y-axis provides the proportion of each county's population in each of 4 age strata. Estimated Spearman's correlation coefficients are provided in each panel. More populous counties have higher proportions of their population comprised of people 20-50 years of age than less populous counties.

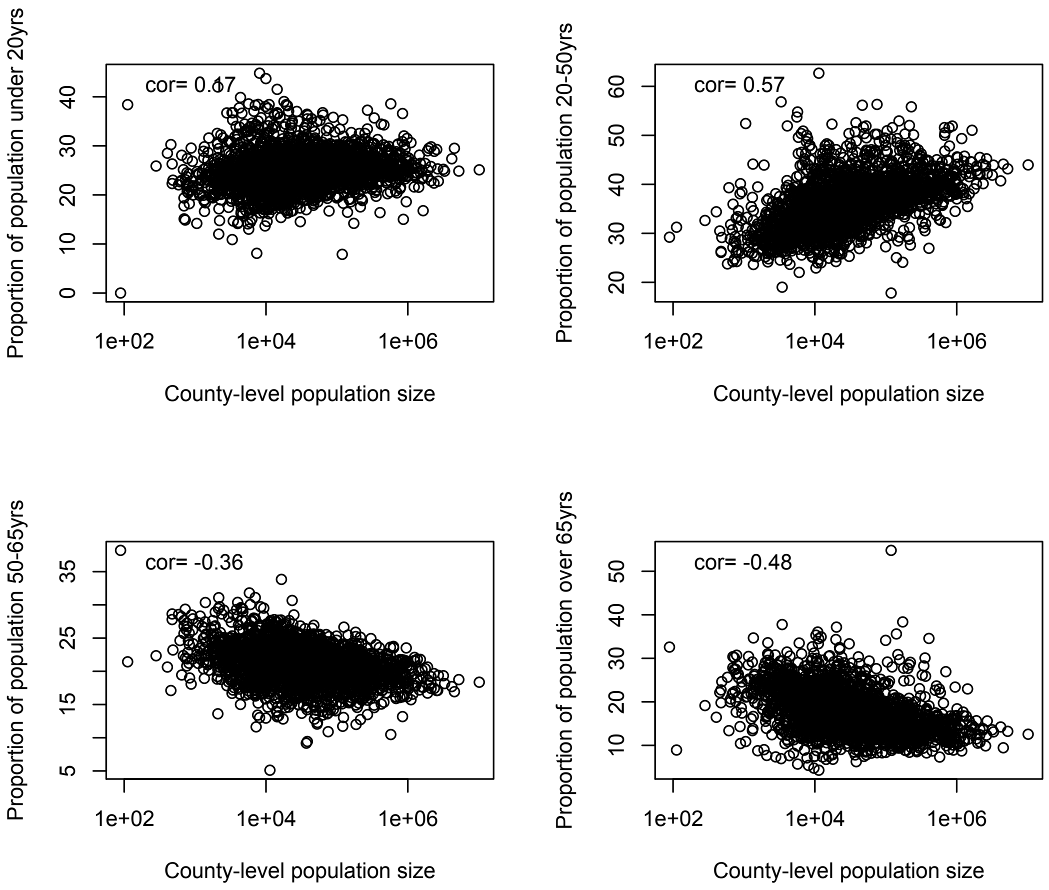

References

1. Viboud C, Bjørnstad ON, Smith DL, Simonsen L, Miller MA, Grenfell BT. Synchrony, waves, and spatial hierarchies in the spread of influenza. Science. 2006 Apr 21;312(5772):447–51.

2. Serfling RE. Methods for current statistical analysis of excess pneumonia-influenza deaths. Public Health Rep. 1963 Jun;78(6):494–506.

3. Gog JR, Ballesteros S, Viboud C, Simonsen L, Bjornstad ON, Shaman J, et al. Spatial Transmission of 2009 Pandemic Influenza in the US. PLoS Comput Biol. 2014 Jun;10(6):e1003635.

4. Viboud C, Charu V, Olson D, Ballesteros S, Gog J, Khan F, et al. Demonstrating the use of high-volume electronic medical claims data to monitor local and regional influenza activity in the US. PloS One. 2014;9(7):e102429.

5. Brockmann D, Helbing D. The hidden geometry of complex, network-driven contagion phenomena. Science. 2013 Dec 13;342(6164):1337–42.

6. BjØrnstad ON, Falck W. Nonparametric spatial covariance functions: Estimation and testing. Environ Ecol Stat. 2001 Mar;8(1):53–70.

7. Diggle PJ. Spatio-temporal point processes, partial likelihood, foot and mouth disease. Stat Methods Med Res. 2006 Aug;15(4):325–36.

8. Diggle PJ, Kaimi I, Abellana R. Partial-likelihood analysis of spatio-temporal point-process data. Biometrics. 2010 Jun;66(2):347–54.

9. CDC. Influenza (flu) including seasonal, avian, swine, pandemic, and other. [Internet]. Centers for Disease Control and Prevention, Available from: http://www.cdc.gov/flu/index.htm
